# Determination of Insoluble, Soluble, and Total Dietary Fiber in Foods Using a Rapid Integrated Procedure of Enzymatic-Gravimetric-Liquid Chromatography: First Action 2022.01

**DOI:** 10.1093/jaoacint/qsac098

**Published:** 2022-08-16

**Authors:** Barry V McCleary, Ciara McLoughlin

**Affiliations:** Fibercarb, Murrumburrah, Eden Rd, Greystones, County Wicklow A63YW01, Ireland; Analytical Department, Neogen Ltd, Bray Business Park, Southern Cross Rd, Bray, County Wicklow A98YV29, Ireland

## Abstract

**Background:**

A simple, accurate, and reliable method for the measurement of total dietary fiber (TDF) according to the Codex definition (2009) was developed and successfully validated as AOAC *Official Method of Analysis* (OMA) **2017.16**. Subsequently, OMA **2017.16** was modified to allow separate measurement of soluble dietary fiber (SDF) and insoluble dietary fiber (IDF) fractions.

**Objective:**

To perform a collaborative study to evaluate the repeatability and reproducibility of OMA **2017.16** modification for the measurement of total dietary fiber (TDF) as IDF and SDF measured as (1) water SDF that precipitates in 78% aqueous ethanol (SDFP), and (2) water SDF that remains soluble in 78% aqueous ethanol (SDFS) of degree of polymerization ≥3.

**Methods:**

Duplicate test portions are incubated with pancreatic α-amylase (PAA), amyloglucosidase (AMG), and protease under the conditions employed in OMA **2017.16**. For the measurement of IDF, the digestate is filtered and the IDF determined gravimetrically. SDFP in the IDF filtrate is precipitated with alcohol and captured by filtration and determined. SDFS in the SDFP filtrate is recovered and quantitated by LC. The matrixes included cereal products and flours, vegetables, health food snacks, soup, chocolate, and beans. Additional materials were analyzed by collaborators as “practice samples”.

**Results:**

With the diethylene glycol internal standard, all multi-laboratotu (MLV) matrixes resulted in repeatability relative standard deviations (RSD_r_) for TDF analyses of <3.60% and RSD_R_ ranging from 4.55 to 9.26%. For the practice samples, the RSD_R_ for TDF ranged from 6.69 to 11.68%.

**Conclusion:**

OMA **2022.01** meets the AOAC requirements for repeatability and reproducibility and the data support First Action status.

**Highlights:**

OMA **2022.01** is a robust and reproducible method for the analysis of insoluble, soluble (SDFP and SDFS), and TDF in a wide range of matrixes.

The definition of dietary fiber adopted by the Codex Alimentarius Commission (CAC) in June 2009 (1) includes carbohydrate polymers that are not hydrolyzed by the endogenous enzymes in the human small intestine, including resistant starch (RS) as well as non-digestible oligosaccharides (where allowed by national authorities). To meet the needs of this definition, a method was developed (2) and validated as AOAC Method **2009.01** (AACC Method 32–45.01) and AOAC Method **2011.25** (AACC Method 32–50.01; 3–7). Subsequently, limitations of this method were identified, including the time of incubation with pancreatic α-amylase (PAA) plus amyloglucosidase ([AMG] 16 h; 8, 9) not being physiologically relevant, excessive hydrolysis and thus underestimation of phosphate cross-linked starch (RS_4_), production of resistant oligosaccharides (10, 11) from non-RS, underestimation of fructo-oligosaccharides (FOS) (11), and the use of sodium azide (a toxic chemical) as a preservative. This led to the development of an improved method for measurement of total dietary fiber (TDF) in which all limitations identified were addressed, namely, the rapid integrated total dietary fiber method (RINTDF; 8), which was successfully validated in an AOAC/AACCI/ICC multilaboratory study, to become AOAC *Official Method of Analysis* (OMA) **2017.16** (12), AACCI recommended method 32–60.01 (13), and ICC method 185.

AOAC Method **2017.16** was then modified (according to the steps described in AOAC Method **2011.25)** to allow the measurement of insoluble dietary fiber (IDF) and soluble dietary fiber (SDF) [as SDF that precipitates in 78% aqueous ethanol (SDFP) plus SDF that remains soluble in 78% aqueous ethanol (SDFS)] summed as TDF. In the study described here, this modified method has been subjected to interlaboratory validation under the auspices of AOAC INTERNATIONAL.

## Multilaboratory Validation Study

### Practice Materials

Prior to the collaborative study, all participating laboratories were provided practice samples to familiarize themselves with the method and to ensure adequate method performance. Laboratories were shipped four matrix samples along with OMA **2022.01**, required enzymes, a control sample, data reporting sheets, and an Excel calculator. Each laboratory was asked to perform a single analysis of each sample, to ask questions regarding the method, and to provide feedback to the method author. The samples included a health snack bar, cookies containing FOS, cauliflower, and whole meal pita bread.

- *Health snack bar and cookies containing FOS.—*Health snack (Nature Valley Peanut Bar) with high fiber content and cookies containing fiber were purchased from a local supermarket and homogenized with a high-speed blender (e.g., Nutri Bullet). Portions of approximately 100 g were transferred to 2 L beakers and approximately 800 mL petroleum ether (or hexane) was added to each and stirred intermittently with a spatula, in a well-ventilated fume hood over 15 min. The solids were allowed to settle over approximately 4 h and the supernatant solution was carefully decanted and discarded. This process was repeated a further two times. The solids were transferred to a flat polypropylene tray and allowed to dry in a well-ventilated fume hood over approximately 3 h and then weighed. The content of fat remaining in the samples was determined using the ANKOM XT15 extractor. The dry material was ground until 100% passed a 0.5 mm sieve and then thoroughly mixed in a plastic bag by inversion and transferred and stored in well-sealed Duran glass bottles at room temperature away from direct sunlight. The health snack bar, defatted as described above, had an original fat content of 25.4%. Subsequent analysis of the dried product using the ANKOM XT15 extractor gave a residual fat content of 6.3%. Cookies containing FOS, extracted as described above, had an original fat content of 21.2%, and subsequent analysis of the dried product using the ANKOM defatting equipment gave a residual fat content of 3.6%.
- *Cauliflower.—*Fresh produce was procured from a local supermarket. The florets were removed and steamed until tender, drained, and cooled to room temperature. The material was chopped finely, weighed, and lyophilized, with wet and dry weights recorded. The recovered dry weight of steamed cauliflower was 11.1%. A sample (approximately 300 g) was ground in a Nutri Bullet homogenizer followed by further grinding in a grinding mill until 100% passed through a 0.5 mm sieve. The ground material was thoroughly mixed in a plastic bag by inversion and transferred and stored in well-sealed Duran glass bottles at room temperature away from direct sunlight.
- *Whole meal pita bread.—*Product was ground to crumbs in a kitchen blender and lyophilized over 2 days and then further ground in a grinding mill until 100% passed through a 0.5 mm sieve. The ground material was thoroughly mixed in a plastic bag by inversion and transferred and stored in well-sealed Duran glass bottles at room temperature away from direct sunlight.
  Test portions of approximately 5 g of each sample type were transferred to pre-labelled glass vials which were sealed with rubber grommets and screw caps. Upon receipt, the collaborators were instructed to store all test portions at room temperature away from direct sunlight until use.
- *Moisture content of practice samples.—*Moisture content of the products used in the study were determined using an Ohaus MB45 moisture analyzer. Values obtained were: health snack bar 9.8%; cookies containing FOS 6.5%; cauliflower 6.1%; whole meal pita bread 2.3%.

### Collaborative Study Materials

Eight pre-prepared foods were selected for the collaborative study to cover a broad range of food categories. These included kidney beans, carrots, dark rye crispbread, barley flour, oat bran, chocolate, soup powder containing dietary fiber, and a health food nutrition bar.

- *Kidney beans (canned and freeze dried).—*Product was purchased from a local supermarket. Canned beans were transferred to a sieve and washed with distilled water to remove all viscous solution. One kg washed beans was lyophilized over 2 days and then ground in a kitchen blender and then a grinding mill until 100% passed through a 0.5 mm sieve. The ground material was collected in a plastic bag, mixed thoroughly by inversion, and then transferred to 1 L Duran bottles, well-sealed and stored at room temperature away from direct sunlight.
- *Carrots (steamed and freeze dried).—*Products were purchased from a local supermarket, peeled, and steamed until tender (approximately15 min), homogenized in a kitchen blender, weighed, transferred to lyophilizer trays and dried over 2 days. The dry material was ground in a grinding mill until 100% passed through a 0.5 mm sieve. The ground material was collected in a plastic bag, mixed thoroughly by inversion, weighed, and then transferred to 1 L Duran bottles, well-sealed and stored at room temperature away from direct sunlight.
- *Dark rye crispbread (Ryvita).—*This product was purchased from a local supermarket and ground in a grinding mill until 100% passed through a 0.5 mm sieve. The ground material was collected in a plastic bag, mixed thoroughly by inversion, and then transferred to 1 L Duran bottles, well-sealed and stored at room temperature away from direct sunlight.
- *Barley MAX flour (high fiber variety).—*Product was obtained from The Healthy Grain Pty Limited, South Yarra, Victoria, Australia. The flour was ground in a grinding mill until 100% passed through a 0.5 mm sieve. The ground material was collected in a plastic bag, mixed thoroughly by inversion, and then transferred to 1 L Duran bottles, well-sealed and stored at room temperature away from direct sunlight.
- *Oat bran.—*Product was purchased from a local supplier, ground in a grinding mill until 100% passed through a 0.5 mm sieve. The ground material was collected in a plastic bag, mixed thoroughly by inversion, and then transferred to 1 L Duran bottles, well-sealed and stored at room temperature away from direct sunlight.
- *Miso soup powder (containing resistant maltodextrins).—*Miso soup powder, containing soluble resistant maltodextrins and seaweed polysaccharide, was obtained from a Japanese supermarket and ground in a grinding mill until 100% passed through a 0.5 mm sieve. The ground material was collected in a plastic bag, mixed thoroughly by inversion, and then transferred to 1 L Duran bottles, well-sealed and stored at room temperature away from direct sunlight.
- *Chocolate and health food nutrition bar (Fiber 1).—*A commercial chocolate product containing Fibersol-2 was obtained from a supermarket in Japan. A health food nutrition bar (Fiber 1 Salted Caramel Bar) was obtained from a local supermarket. Products were homogenized with a high-speed blender (e.g., Nutri Bullet). Portions of approximately 100 g were transferred to 2 L beakers and approximately 800 mL petroleum ether (or hexane) was added to each and stirred intermittently with a spatula, in a well-ventilated fume hood over 15 min. The solids were allowed to settle over approximately 4 h and the supernatant solution was carefully decanted and discarded. This process was repeated a further two times. The solids were transferred to a flat polypropylene tray and allowed to dry in a well-ventilated fume hood over approximately 3 h and then weighed. The content of fat remaining in the samples was determined using the ANKOM XT15 extractor. The dry material was ground until 100% passed a 0.5 mm sieve and then thoroughly mixed in a plastic bag and transferred and stored in well-sealed Duran glass bottles at room temperature away from direct sunlight. Chocolate, defatted as described above, had an original fat content of 36.2%. Subsequent analysis of the dried product using the ANKOM XT15 extractor gave a residual fat content of 6.5%. The health food bar had an original fat content of 15.4%. Subsequent analysis of the dried product using the ANKOM XT15 extractor gave a residual fat content of 0%.
- *Moisture content of collaborative study samples.—*Moisture content was determined using an Ohaus MB45 moisture analyzer. Values obtained were: kidney beans 1.2%, dried carrots 4.1%, dark rye crispbread 4.1%, barley MAX flour 6.9%, oat bran 9.9%, Miso soup powder 7.0%, defatted chocolate containing fiber 6.4%, and defatted Fiber 1 salted caramel bar 3.7%.
  Test portions of approximately 5 g of each sample type were transferred to pre-labelled glass vials which were sealed with rubber grommets and screw caps. Two randomly selected test portion vials from each matrix preparation were packaged for shipment. Samples, anion and cation exchange resins, 15 mL polypropylene tubes plus caps, a copy of the method, a link to a video of the method, Excel-based data report forms, Mega-Calc™ Data Calculator (Megazyme, Bray, Ireland), sample storage instructions, and an adequate supply of enzymes in the Rapid Integrated TDF assay kit (K-RINTDF), and details on how to prepare and store these, were distributed to collaborating laboratories by express overnight shipment. Upon receipt, the collaborators were instructed to store all test portions at room temperature away from direct sunlight until the start of the study and to store kit components as described on the individual bottle labels.

### Statistical Treatment

Results were submitted by collaborators using supplied Excel-based spreadsheets and evaluated according to AOAC guidelines using an AOAC statistical workbook. Outlier results identified by the Cochran’s test for extremes of repeatability and the Grubb’s test for extremes of reproducibility were omitted from further calculations. Also determined were repeatability (s_r_) and reproducibility (s_R_) standard deviations, relative standard deviations of repeatability (RSD_r_) and reproducibility (RSD_R_), and measurement uncertainty (*MU*) values.

**AOAC *Official Method*^SM^ 2022. 01**

**Insoluble, Soluble, and Total Dietary Fiber**

**in Foods and Food Ingredients**

**Rapid Integrated Enzymatic-Gravimetric-**

**Liquid Chromatography**

**First Action 2022**

[Applicable to plant material, foods, and food ingredients consistent with CAC Definition adopted in 2009 and modified slightly in 2010 (ALINORM 09/32/REP and ALINORM 10/33/REP, respectively) including naturally occurring, isolated, modified, and synthetic carbohydrate polymers and oligosaccharides meeting that definition. This method is specifically designed for the analysis of foods “as eaten”.]

*Caution*: Solvents employed are common-use solvents and reagents. Refer to appropriate manuals or safety data sheets to ensure that the safety guidelines are applied before using chemicals. Store in a flammable liquid storage cabinet. Harmful if inhaled, swallowed, or absorbed through the skin. Use appropriate personal protective equipment such as a lab coat, safety glasses, rubber gloves, and fume hood. Dispose of all materials according to federal, state, and local regulations.

*See* Tables **2022.01A** **–** **C** for S_r_, S_R_, RSD_r_, and RSD_R_ for insoluble, soluble, and TDF with glycerol internal standard.

**Table 2022.01A.** Interlaboratory study results for IDF in foods (RINTDF Method—glycerol internal standard) in which outlier data from Laboratories 1, 8, 9, and 11 (*see*Table 3) were excluded; statistical evaluation according to AOAC statistics format

Sample/parameter	A & F^a^	B & J^b^	C & M^c^	D & L^d^	E & P^e^	G & H^f^	I & O^g^	K & N^h^
No. of Labs	16	16	13	15	15	15	16	15
Mean, g/100 g	19.89	12.61	7.28	13.33	12.40	23.00	5.64	11.97
S_r_	0.874	0.346	0.532	0.620	0.544	0.889	0.762	0.203
S_R_	1.430	0.728	1.050	0.972	1.402	1.472	1.993	0.393
RSD_r_	4.38	2.74	7.13	4.65	4.41	3.86	13.52	1.70
RSD_R_	7.18	5.77	14.42	7.29	11.36	6.40	34.10	3.28

a A & F = Kidney beans (canned and freeze dried).

b B & J = Ryvita dark rye crispbread.

c C & M = Chocolate with added fiber.

d D & L = Steamed carrots (freeze dried).

e E & P = Oat bran.

f G & H = Barley MAX flour (high fiber variety).

g I & O = Miso soup powder containing resistant maltodextrins and seaweed.

h K & N = Nutrition bar (Fiber 1).

**Table 2022.01B.** Interlaboratory study results for SDF in foods (RINTDF Method—glycerol internal standard) in which outlier data from laboratories 4, 6, and 9 were excluded (*see*Table 3); statistical evaluation according to AOAC statistics format

Sample/parameter	A & F^a^	B & J^b^	C & M^c^	D & L^d^	E & P^e^	G & H^f^	I & O^g^	K & N^h^
No. of Labs	16	15	15	16	16	16	14	14
Mean, g/100 g	7.41	10.08	23.83	10.53	10.80	18.19	33.25	13.39
S_r_	0.368	0.362	1.009	0.533	0.575	0.464	0.918	0.357
S_R_	0.823	0.813	2.535	0.906	1.080	2.129	3.123	1.821
RSD_r_	4.97	3.59	4.24	5.06	5.33	2.55	2.76	2.67
RSD_R_	11.23	8.82	10.64	8.61	10.00	11.70	9.40	13.60

a A & F = Kidney beans (canned and freeze dried).

b B & J = Ryvita dark rye crispbread.

c C & M = Chocolate with added fiber.

d D & L = Steamed carrots (freeze dried).

e E & P = Oat bran.

f G & H = Barley MAX flour (high fiber variety).

g I & O = Miso soup powder containing resistant maltodextrins and seaweed.

h K & N = Nutrition bar (Fiber 1).

**Table 2022.01C.** Interlaboratory study results for TDF in foods (RINTDF Method glycerol internal standard) in which outlier data from laboratories 4, 9, 11, and 12 (*see*Table 3) were excluded; statistical evaluation according to AOAC statistics format

Sample/parameter	A & F^a^	B & J^b^	C & M^c^	D & L^d^	E & P^e^	G & H^f^	I & O^g^	K & N^h^
No. of Labs	16	15	16	14	16	16	15	15
Mean, g/100 g	27.07	22.58	31.34	23.79	23.11	40.85	40.44	26.29
S_r_	0.759	0.560	1.409	0.697	0.816	1.055	1.586	0.373
S_R_	1.395	1.132	2.281	1.185	2.002	2.020	4.351	2.988
RSD_r_	2.80	2.48	4.50	2.93	3.53	2.58	4.03	1.42
RSD_R_	5.16	5.01	7.28	4.98	8.67	4.94	11.06	11.37

a A & F = Kidney beans (canned and freeze dried).

b B & J = Ryvita dark rye crispbread.

c C & M = Chocolate with added fiber.

d D & L = Steamed carrots (freeze dried).

e E & P = Oat bran.

f G & H = Barley MAX flour (high fiber variety).

g I & O = Miso soup powder containing resistant maltodextrins and seaweed.

h K & N = Nutrition bar (Fiber 1).

*See* Tables **2022.01D** **–** **F** for diethylene glycol (DEG) internal standard.

**Table 2022.01D.** Interlaboratory study results for IDF in foods (RINTDF Method—DEG internal standard) in which outlier data from Laboratories 5, 8, 9, and 11 (*see*Table 4) were excluded; statistical evaluation according to AOAC statistics format

Sample/parameter	A & F^a^	B & J^b^	C & M^c^	D & L^d^	E & P^e^	G & H^f^	I & O^g^	K & N^h^
No. of Labs	14	15	13	14	14	13	15	14
Mean, g/100 g	19.89	12.64	7.09	13.26	12.32	22.91	5.37	11.98
S_r_	0.861	0.406	0.532	0.527	0.562	0.606	0.769	0.153
S_R_	1.471	0.759	0.970	0.929	1.551	1.455	1.931	0.350
RSD_r_	4.33	3.21	7.50	3.97	4.60	2.65	14.33	1.28
RSD_R_	7.40	6.00	13.68	7.00	12.69	6.35	35.97	2.93

a A & F = Kidney beans (canned and freeze dried).

b B & J = Ryvita dark rye crispbread.

c C & M = Chocolate with added fiber.

d D & L = Steamed carrots (freeze dried).

e E & P = Oat bran.

f G & H = Barley MAX flour (high fiber variety).

g I & O = Miso soup powder containing resistant maltodextrins and seaweed.

h K & N = Nutrition bar (Fiber 1).

**Table 2022.01E.** Interlaboratory study results for SDF in foods (RINTDF Method—DEG internal standard) in which outlier data from Laboratories 9 and 13 were excluded (*see*Table 4); statistical evaluation according to AOAC statistics format

Sample/parameter	A & F^a^	B & J^b^	C & M^c^	D & L^d^	E & P^e^	G & H^f^	I & O^g^	K & N^h^
No. of Labs	14	15	15	15	15	14	14	14
Mean, g/100 g	7.64	10.36	24.60	10.72	10.97	18.46	34.94	25.20
S_r_	0.419	0.409	0.555	0.533	0.578	0.710	0.836	0.689
S_R_	0.834	0.779	2.240	0.879	1.098	2.231	2.636	2.940
RSD_r_	5.43	3.95	2.26	4.97	5.28	3.85	2.40	2.73
RSD_R_	10.81	8.35	9.11	8.19	10.03	12.08	7.56	11.99

a A & F = Kidney beans (canned and freeze dried).

b B & J = Ryvita dark rye crispbread.

c C & M = Chocolate with added fiber.

d D & L = Steamed carrots (freeze dried).

e E & P = Oat bran.

f G & H = Barley MAX flour (high fiber variety).

g I & O = Miso soup powder containing resistant maltodextrins and seaweed.

h K & N = Nutrition bar (Fiber 1).

**Table 2022.01F.** Interlaboratory study results for TDF in foods (RINTDF Method DEG internal standard) in which outlier data from Laboratories 2 and 12 were excluded (*see*Table 4); statistical evaluation according to AOAC statistics format

Sample/parameter	A & F^a^	B & J^b^	C & M^c^	D & L^d^	E & P^e^	G & H^f^	I & O^g^	K & N^h^
No. of Labs	14	15	15	14	15	14	14	14
Mean, g/100 g	27.33	22.87	31.60	23.89	23.24	41.19	40.40	37.07
S_r_	0.705	0.588	0.618	0.706	0.828	1.008	1.346	0.586
S_R_	1.570	1.106	2.104	1.120	2.146	1.872	3.049	2.917
RSD_r_	2.58	2.57	1.96	2.95	3.57	2.45	3.34	1.58
RSD_R_	5.74	4.63	6.66	4.68	9.26	4.55	8.27	7.87

a A & F = Kidney beans (canned and freeze dried).

b B & J = Ryvita dark rye crispbread.

c C & M = Chocolate with added fiber.

d D & L = Steamed carrots (freeze dried).

e E & P = Oat bran.

f G & H = Barley MAX flour (high fiber variety).

g I & O = Miso soup powder containing resistant maltodextrins and seaweed.

h K & N = Nutrition bar (Fiber 1).

### A. Principle

The method measures IDF, SDF and TDF as defined by the CAC (1). The method quantitates IDF and SDF which precipitates in 78% aqueous ethanol (SDFP) by gravimetric procedures, SDF which remains soluble in 78% aqueous ethanol (SDFS) by HPLC, and TDF by gravimetric and HPLC procedures (Figure **2022.01A**). SDF is calculated by combining the weights of SDFP and SDFS. RS is captured in the IDF fraction. The method combines key attributes of OMA **985.29, 2001.03**, **2011.25**, and **2017.16**. Duplicate test portions are incubated with PAA and AMG for 4 h at 37°C in sealed 250 mL bottles in a shaking water bath while mixing in orbital motion or stirring with a magnetic stirrer, during which time non-RS is solubilized and hydrolyzed to glucose and maltose by the combined action of the two enzymes. The reaction is terminated by pH adjustment followed by temporary heating. Protein in the sample is digested with protease. For the separate measurement of IDF and SDF, the sample suspension is filtered and the filtrate recovered. IDF is captured on a sintered glass crucible, washed with ethanol (EtOH) or industrial methylated spirits (IMS) and acetone, dried and weighed. With all gravimetric determinations, one of the duplicate residues is analyzed for protein, the other for ash and these weights are subtracted from the residue weights. To the filtrate, ethanol is added to a concentration of 78%, and the precipitated SDFP is captured on a sintered glass crucible, washed with ethanol and acetone, dried, and weighed. SDFS in the filtrate is concentrated, deionized with resins, and quantitated by HPLC. Using this method, TDF = IDF + SDFP + SDFS. For measurement of TDF directly, use AOAC OMA **2017.16**. The current method differs from OMA **2011.25** in that incubation time with PAA and AMG is reduced from 16 to 4 h (with higher concentrations of enzymes used) to better simulate human intestinal residence time. Improved deionization and HPLC separation of SDFS is incorporated, glycerol or DEG is used as the internal standard, and potentially hazardous sodium azide is removed from the incubation buffer.

**Figure 2022.01A.**
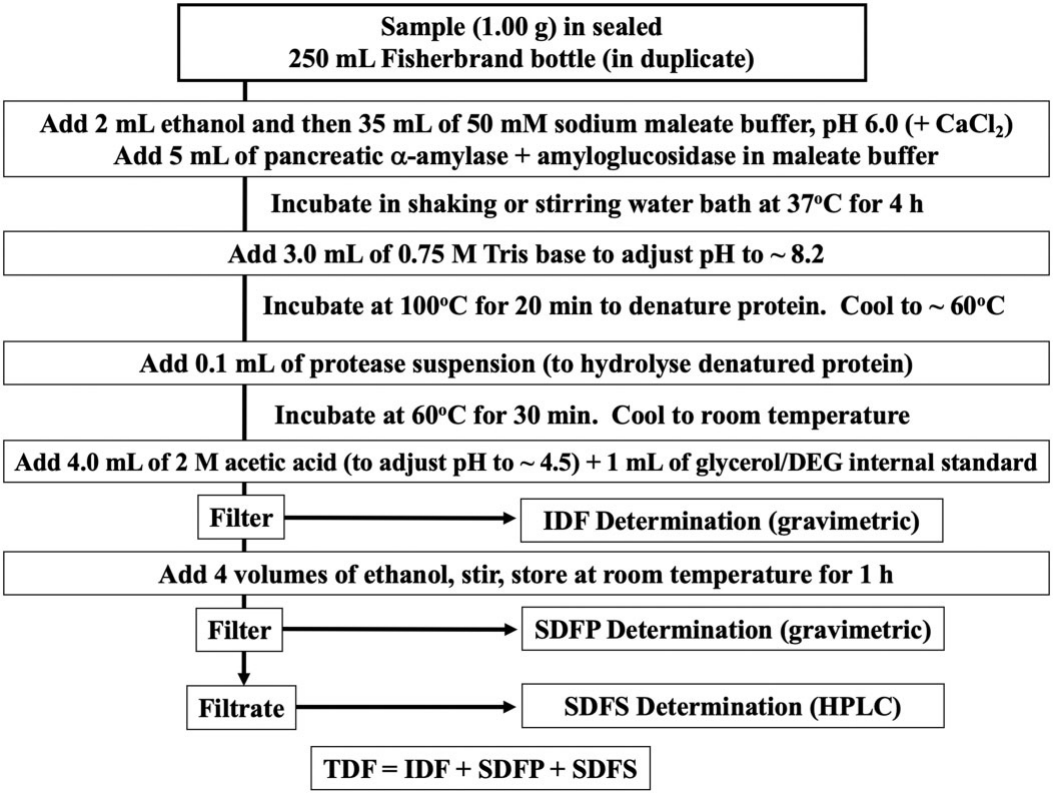
Rapid integrated TDF method showing separation of IDF, SDFP, and SDFS fractions.

### B. Chemicals and Reagents

- *EtOH 95% (v/v) or IMS.—*IMS made up of EtOH 84.8333% (w/w), 85.952% (v/v); water 5.6571% (w/w), 4.524% (v/v); 2-propanol 4.9118% (w/w), 5.0000% (v/v); methanol 4.5979% (w/w), 4.524% (v/v). It can be prepared by mixing 5 volumes 2-propanol with 95 volumes denatured ethanol formula SDA-3A (100 volumes 95% EtOH combined with 5 volumes methanol).
- *EtOH or IMS, 78%.—*Place 179 mL water into 1 L volumetric flask. Dilute to volume with 95% EtOH or IMS. Mix.
- *Acetone*.—Reagent grade.
- *Stock PAA plus AMG powder.—*PAA (40 KU/g) plus AMG (17 KU/g) as a freeze-dried powder mixture. [*Note:* One unit AMG activity is the amount of enzyme required to release one µmole d-glucose from soluble starch per minute at 40°C and pH 4.5; one unit PAA activity is the amount of enzyme required to release one µmole *p*-nitrophenyl from Ceralpha reagent per minute at 40°C and pH 6.9; OMA **2002.01**. PAA/AMG preparations should be essentially devoid of β-glucanase, β-xylanase, and detectable levels of free d-glucose. Stable for ∼4 years at −20°C.
- *PAA (4 KU/5* *mL)/AMG (1.7 KU/5* *mL).—*Immediately before use, dissolve 1 g PAA/AMG powder, **B(d)**, in 50 mL sodium maleate buffer (50 mM, pH 6.0 plus 2 mM CaCl_2_) and stir for approximately 5 min. Store on ice during use. Use on the day of preparation. *Alternatively:* Some individuals are allergic to powdered PAA and/or AMG. In this instance, engage an analyst who is not allergic to prepare the powdered enzymes as an ammonium sulphate suspension as follows: Gradually add 5 g PAA/AMG powder mix [PAA 40 KU/g plus AMG 17 KU/g; **B(d)**] to 70 mL cold, distilled water in a 200 mL beaker on a magnetic stirrer in a laboratory hood and stir until the enzymes are completely dissolved (approximately 5 min). Add 35 g granular ammonium sulphate and dissolve by stirring. Adjust the volume to 100 mL with ammonium sulphate solution (50 g/100 mL) and store at 4°C. This preparation contains PAA at 2 KU/mL and AMG at 0.85 KU/mL. Stable at 4°C for 3 months.
- *Protease suspension (50* *mg/mL, approximately 6 tyrosine U/mg).—*Stabilized suspension in 3.2 M ammonium sulphate. Swirl gently before use. Dispense using a positive displacement dispenser. Protease must be devoid of α-amylase and essentially devoid of β-glucanase and β-xylanase. Use as supplied. Stable for ∼4 years at 4°C.
- *Glycerol internal standard (100* *mg/mL in 0.02%, w/v, sodium azide).—*Supplied in the Rapid Integrated TDF kit (Cat. No, K-RINTDF; Megazyme Ltd, Bray, Ireland). Stable for ∼4 years at 4°C.
- *Diethyleneglycol (DEG) internal standard (100* *mg/mL).—*Carefully weigh 10.00 g of diethylene glycol into a 100 mL beaker on an analytical balance. Remove the beaker from the balance and add ∼30 mL of aqueous sodium azide solution (0.02%, w/v), **B(p).** Transfer the solution to a 100 mL volumetric flask (using a funnel). Wash the beaker with approximately 2 × 20 mL of the aqueous sodium azide solution to remove all DEG and transfer this to the volumetric flask. Adjust the volume to 100 mL with aqueous sodium azide solution, **B(p)**. Transfer the solution to a 100 mL Duran bottle and store at room temperature in the dark. Stable for ∼6 months stored in the dark at room temperature.
- *LC retention time standard (maltodextrins).—*Dissolve 1.25 g retention time standard [supplied in the Rapid Integrated TDF kit (Cat. No, K-RINTDF; Megazyme Ltd)] consisting of corn syrup solids [degree of polymerization (DP) ≥3] and maltose in 30 mL of 0.02% sodium azide solution, **B(p)**, and transfer to a 50 mL volumetric flask. Add 5 mL glycerol internal standard, **B(g)**. Bring to 50 mL with 0.02% sodium azide solution, **B(p)**. Transfer solutions to 50 mL Duran bottle. Stable at 4°C for ∼2 years.
- *d-Glucose/glycerol LC standard (10* *mg/mL each in 0.02% w/v sodium azide).—*Supplied in the Rapid Integrated TDF kit (Cat. No, K-RINTDF; Megazyme Ltd). Stable for ∼4 years at 4°C.
- *d-Glucose/DEG LC standard (10* *mg/mL each).—*Transfer 1.00 g diethylene glycol into a 100 mL beaker on an analytical balance. Remove the beaker from the balance and add ∼30 mL aqueous sodium azide (0.02% w/v), **B(p)**, and mix well. Separately, weigh 1.00 g dried glucose into a separate 100 mL beaker on an analytical balance. Remove the beaker from the balance and add ∼30 mL aqueous sodium azide (0.02% w/v) and mix well. Transfer both solutions to a 100 mL volumetric flask and use aqueous sodium azide solution (0.02% wv) **B(p)** to completely rinse the beakers and transfer the washings into the volumetric flask (using a funnel). Adjust the final volume to 100 mL and mix the contents well. Transfer the solution to a 100 mL Duran bottle and store at room temperature in the dark. Stable for ∼6 months stored in the dark at room temperature.
- *Sodium maleate buffer (50 mM, pH 6.0, with 2 mM CaCl_2_).—*Dissolve 11.6 g maleic acid in 1600 mL deionized water and adjust the pH to 6.0 with 4 M (160 g/L) NaOH solution. Add 0.6 g calcium chloride (CaCl_2_·2H_2_O) and adjust volume to 2 L. Stable for approximately 2 weeks at 4°C.
- *2-(N-morpholino) ethanesulfonic acid (MES) buffer (50mM, pH 6.0, with 2 mM CaCl_2_).—*This can be used as an alternative to sodium maleate buffer, **B(l)**. Dissolve 19.5 g MES in 1600 mL deionized water and adjust the pH to 6.0 with 4 M (160 g/L) NaOH solution. Add 0.6 g calcium chloride (CaCl_2_·2H_2_O) and adjust volume to 2 L. Solution is stable for approximately 2 weeks at 4°C.
- *Tris base (0.75 M).—*Add 90.8 g Tris base to approximately 800 mL distilled water and dissolve. Adjust to pH 11.0. Adjust volume to 1 L. Stable for ∼1 year at room temperature.
- *Acetic acid solution (2 M).—*Add 115 mL glacial acetic acid (Fluka 45731; Sigma-Aldrich Ireland Ltd) to a 1 L volumetric flask. Dilute to 1 L with distilled water. Stable for ∼1 year at room temperature.
- *Sodium azide solution (0.02%, w/v).—*Add 0.2 g sodium azide to 1 L deionized water and dissolve by stirring. Stable at room temperature for ∼1 year.
- *Cleaning solution.—*Micro (International Products Corp., Trenton, NJ, USA). Make a 2% solution with deionized water.
- *pH standards.—*Certified buffer solutions at pH 4.0, 7.0, and 10.0.
- *Deionized water*.
- *Celite.—*Acid-washed, pre-ashed. (Cat. No. G-CEL100 or G-CEL500, Megazyme Ltd; or Cat. No. C8656, Sigma-Aldrich Ireland Ltd).
- *Amberlite^®^ FPA53 (OH^−^) resin and Ambersep^®^ 200 (H^+^) resin.—*Ion exchange capacity ≥1.6 meq/mL each (Cat. Nos. G-AMBH and G-AMBOH, Megazyme Ltd).

Items **(d)**, **(f)**, **(g)**, **(i)**, and **(j)** are supplied in the Rapid Integrated TDF kit (Cat. No, K-RINTDF; Megazyme Ltd, Bray, Ireland), but preparations of reagents and buffers that meet the criteria as specified in the method may also be used. Solutions of DEG (100 mg/mL), **B(h),** and the d-glucose/DEG LC standard solution, **B(k),** must be prepared fresh every 6 months.

### C. Apparatus

- *Grinding mill.—*Centrifugal, with 12-tooth rotor and 0.5 mm sieve, or similar device. Alternatively, cyclone mill can be used for small-test laboratory samples provided they have sufficient air flow or other cooling to avoid overheating samples.
- *Digestion bottles.—*250 mL Fisherbrand^®^ Clear, graduated glass bottles with white polypropylene caps (Cat. No. 16154377; Fisher Scientific, Göteborg, Sweden); Figures **2022.01B** and 2022.01C.
- *Fritted crucible.—*Gooch fritted disk, Pyrex^®^ 50 mL, pore size coarse, American Society for Testing and Materials 40–60 μm (Product No. 32940-50C; Corning Life Sciences, Tewksbury, MA, USA). Prepare four for each sample as follows: ash overnight at 525°C in muffle furnace. Cool furnace to 130°C before removing crucibles to minimize breakage. Remove any residual Celite and ash material by using a vacuum. Soak in 2% Micro cleaning solution, **B**(**q**), at room temperature for 1 h. Rinse crucibles with water and deionized water. For final rinse, use 15 mL acetone and air dry. Add approximately 1.0 g Celite, **B**(**t**), to dried crucibles and dry at 130°C to constant weight. Cool crucible in desiccators for approximately 1 h and record mass of crucible containing Celite.
- *Filtering flask.*—Heavy-walled, 1 L with side arm.
- *Rubber ring adaptors*.—For use to join crucibles with filtering flasks.
- *Vacuum source*.—Vacuum pump or aspirator with regulator capable of regulating vacuum.
- *Water bath(s)*.—Rotary motion, shaking, large-capacity (20–24 L) with covers; capable of maintaining temperatures of 37 ± 1 and 60 ± 1°C; equipped with automatic timers for on–off operation (Grant Instruments, Shepreth, Royston, United Kingdom). Ensure that shaking action/sample agitation in the water bath used is sufficient to maintain sample solids in suspension and no residue build-up or rings of sample material form in the digestion bottle during the enzymatic digestions (i.e., at 150 rev/min; Figure **2022.01B**). A linear motion (back-and-forth shaker) can be used if the bottles are placed at 45° angle in the direction of motion to ensure adequate agitation (if the bottles are vertical or horizontal there will not be sufficient agitation to ensure that the sample remains suspended). Alternatively, a 2mag Mixdrive 15^®^ submersible magnetic stirrer (2mag AG, Munich, Germany) can be used in a water bath maintained at 37 ± 1°C with a circulating heater (Julabo GmbH, Seelbach, Germany). Samples are stirred in digestion bottles, **C**(**b**)*,* with a 7 × 30 mm stir bar, set at about 170 revolutions per minute (rpm) (Figure **2022.01C**).
- *Balance*.—0.1 mg readability, accuracy, and precision.
- *Ovens*.—Two, mechanical convection, set at 103 ± 2 and 130 ± 3°C.
- *Timer.*
- *Desiccator*.—Airtight, with SiO_2_ or equivalent desiccant. Desiccant dried biweekly overnight in 130°C oven, or more frequently as needed.
- *pH meter.*
- *Pipettors and tips.—*50–200 µL and 5 mL capacity.
- *Dispensers.—(* 1 *)* 15 ± 0.5 mL for 78% EtOH (or IMS), 95% ethanol (or IMS), and acetone. *(*2*)* 35 ± 0.5 mL for buffer.
- *Cylinder*.—Graduated, 100 and 500 mL.
- *Magnetic stirrers and stirring bars.*
- *Rubber spatulas.*
- *Muffle furnace*.—525 ± 5°C
- *Polypropylene tubes.—*15 mL, 101 × 16.5 mm, flat base, with screw cap.
- *HPLC system.—*With oven to maintain a column temperature of 80°C and a 50 µL injection loop. Column operating conditions: temperature, 80°C; mobile phase, distilled water; flow rate, 0.5 mL/min.
- *HPLC columns.* Two TSKgel^®^ G2500PW_XL_ columns, 30 cm × 7.8 mm, connected in series. Operated at 80°C. Mobile phase: distilled water at 0.5 mL/min. System must be capable of separating maltose from maltotriose (Figure **2022.01D**). Run time of 60 min to ensure that all materials from the injection are cleared from the column prior to the next injection. If samples contain high levels of either lactose or isomaltose (as seen from HPLC chromatograms) it may be necessary to incubate samples with a mixture of β-galactosidase and oligo-α-1,6-glucosidase before HPLC analysis [*see* **I(g)** *Note*].
- *Cation and anion exchange guard column (containing deionizing/desalting cartridges).—*Cation and anion exchange guard cartridges, H^+^ and ${\text{CO}}_{2}^{3-}$ forms, respectively (Cat. No. 125-0118; Bio-Rad Laboratories, Hercules, CA, USA; includes one cation and one anion cartridge), with guard column holder (Cat. No. 125-039; Bio-Rad Laboratories) to hold the two guard cartridges in series, cation cartridge preceding anion cartridge.
- *Guard column (or pre-column).—*TSKgel PW_XL_ guard column (TOSOH Corp., Tokyo, Japan).
- *Detector.—*Refractive index (RI); maintained at 50°C.
- *Data integrator or computer.—*For peak area measurement.
- *Filters for disposable syringe.—*Polyvinylidene fluoride membrane, 0.45 µm pore size, 13 or 25 mm.

**(aa)** *Filters for water.—*Polyvinylidene fluoride, pore size 0.45 µm, 47 mm.
**(bb)** *Filter apparatus.—*To hold **C(aa)** to filter large volumes of water.
**(cc)** *Syringes.—*10 mL, disposable plastic.
**(dd)** *Syringes.—*Hamilton 100 µL, 710SNR syringe (Sigma-Aldrich Ireland Ltd).
**(ee)** *Rotary evaporator*.—Heidolph Laborota 4000 or equivalent (Heidolph, Elk Grove Village, IL, USA) set at 60°C.
**(ff)** *Thermometer*.—Capable of measuring to 100°C.

**Figure 2022.01B.**
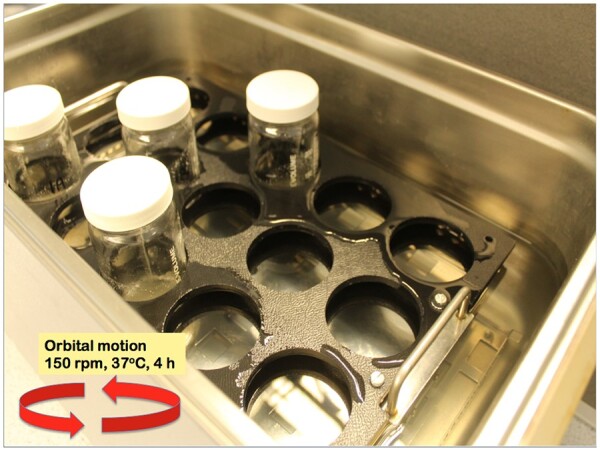
Incubation of samples in Fisherbrand incubation bottles in a shaking water bath showing custom-made polypropylene bottle holder.

**Figure 2022.01C.**
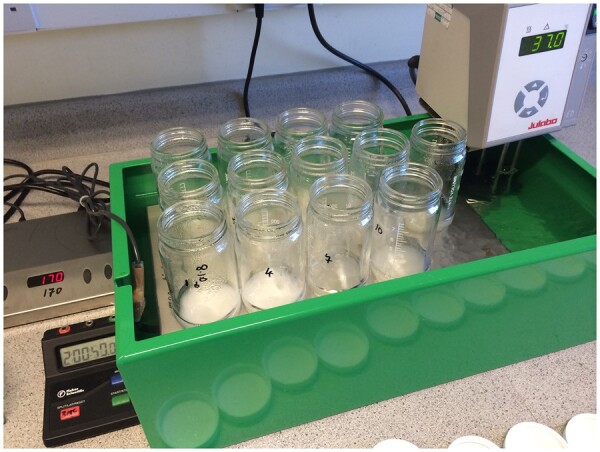
2mag Mixdrive 15 submersible magnetic stirrer in custom-built bath with Fisherbrand incubation bottles.

**Figure 2022.01D.**
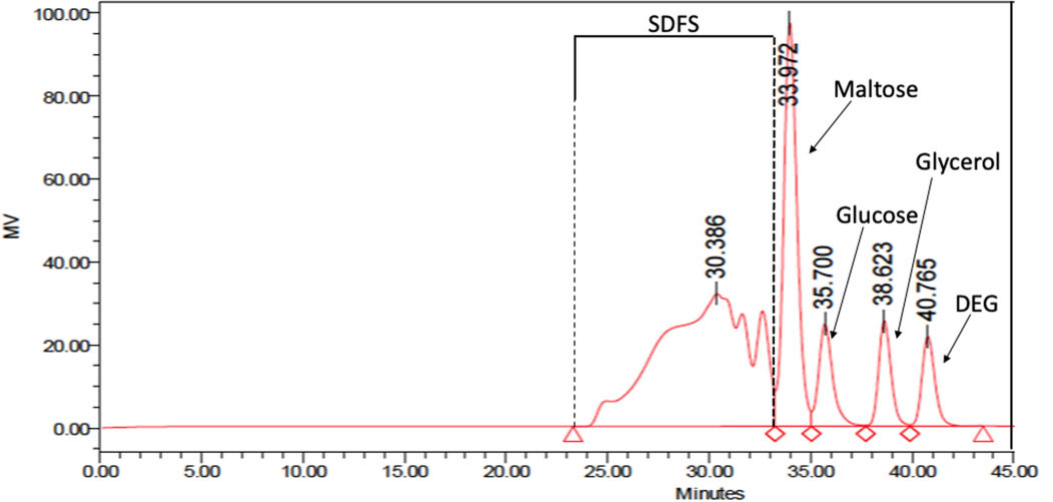
Chromatograms of a mixture of maltodextrins, glucose, glycerol, and diethylene glycol (DEG) on two TSKgel G2500PW_XL_ columns in series. Solvent: distilled water; flow rate: 0.5 mL/min; and temperature: 80°C. Dashed lines show demarcation between DP 2 (maltose) and DP 3 (higher maltodextrins). The fraction shown as SDFS denotes the fraction that would be collected as SDFS; however, in this case, these are maltodextrins that would be hydrolyzed by the PAA/AMG mixture.

### D. Preparation of Test Samples

Collect and prepare samples as intended to be eaten, i.e., baking mixes should be prepared and baked, pasta should be cooked, etc. Defat as per AOAC OMA **985.29** if >10% fat. For high-moisture samples (>25%), it may be desirable to freeze-dry. Grind ca 50 g in a grinding mill, **C(a)**, to pass a 0.5 mm sieve. Transfer all material to a wide-mouthed plastic jar, seal, and mix well by shaking and inversion. Store in the presence of a desiccant.

### E. Enzyme Purity

To ensure absence of undesirable enzymatic activities and effectiveness of desirable enzymatic activities, analyze the standards listed in Table AOAC **991.43B**, (citrus pectin, arabinogalactan, β-glucan, wheat starch, corn starch, casein, and Hylon VII) [Table 2, Reference (8)] each time enzyme lot changes or at a maximum 6 month interval.

**Table 1.** Study data for practice sample evaluation of the RINTDF method for soluble (SDF), insoluble (IDF), and TDF

	P1^a^			P2^b^			P3^c^			P4^d^		
Lab	SDF	IDF	TDF	SDF	IDF	TDF	SDF	IDF	TDF	SDF	IDF	TDF
1	9.00	5.37	14.40	4.01	24.53	28.54	4.65	3.54	8.19	2.99	11.60	14.60
2	10.26	4.42	14.68	5.10	27.20	32.20	4.20	4.12	8.40	3.30	10.90	14.20
3	10.26	4.42	14.70	4.20	22.89	27.09	3.92	3.32	7.24	3.29	11.15	14.43
4	13.64	4.20	17.84	4.83	24.21	29.04	5.43	3.73	9.17	5.25	10.71	15.96
5	15.06	4.66	19.71	6.76	20.10	26.86	4.89	3.91	8.80	3.73	10.82	14.54
6	13.67	4.15	17.80	3.71	22.11	25.82	4.58	3.25	7.83	3.78	10.80	14.58
7	9.73	4.94	14.70	4.25	25.75	30.00	4.16	3.68	7.84	3.23	11.60	14.83
8	11.98	4.73	16.71	5.90	23.02	28.92	3.61	3.52	7.13	3.62	10.72	14.34
9	13.06	3.88	16.94	4.21	22.78	26.99	4.13	3.24	7.37	4.73	9.52	14.25
10	8.60	4.49	13.09	4.35	23.48	27.83	3.80	3.68	7.48	2.79	11.32	14.10
11	10.48	4.39	14.90	8.99	23.34	32.33	2.43	4.12	6.55	0.19	9.68	9.88
12	10.60	4.09	14.69	1.92	26.18	28.10	2.83	3.20	6.03	2.59	10.49	13.08
13	9.96	6.58	16.55	7.34	23.46	30.80	4.96	3.58	8.54	4.19	10.86	15.05
14	9.24	4.98	14.22	3.97	24.39	28.36	4.09	3.39	7.48	3.01	10.78	13.79
15	8.96	4.40	13.35	6.30	23.20	29.50	4.27	3.33	7.59	5.56	10.66	16.22
16	9.55	4.43	13.98	4.24	23.21	27.46	4.15	3.79	7.94	3.63	11.26	14.89
17	12.37	4.37	16.70	4.67	21.50	26.17	3.88	3.50	7.38	3.05	10.62	13.67
Mean, g/100 g	10.97	4.62	15.59	4.99	23.61	28.59	4.12	3.58	7.70	3.47	10.79	14.26
s_R_, g/100 g	1.95	0.62	1.82	1.64	1.71	1.91	0.73	0.29	0.79	1.19	0.56	1.36
RSD_R_, %	17.80	13.45	11.68	32.94	7.25	6.69	17.73	8.03	10.19	34.28	5.16	9.54

a Health snack with high FOS.

b Cauliflower.

c Defatted cookies containing FOS.

d Whole meal pita bread.

### F. Enzymatic Digestion of Sample

- *Blanks.—*With each new lot of enzymes, **B(d)** and **B(f)**, analyze two blanks (containing everything except test portion), along with samples to measure any contribution from reagents to residue. Blank determinations need to be performed just once for each new lot of enzymes (lot number on the vial of enzyme)—it is not necessary to run blank analyses with each set of assays.
- *Samples.—*
  1. Weigh duplicate 1.000 ± 0.005 g samples accurately into 250 mL digestion bottles, **C(b)**.
  2. *Addition of buffer and equilibration.—*Wet the sample with 2.0 mL 95% EtOH or IMS and add 35 mL 50 mM sodium maleate buffer, **B(l)**, or MES buffer, **B(m)**, and a 7 × 30 mm stirrer bar to each bottle. Place bottles on a 2mag Mixdrive 15 magnetic stirrer apparatus in a water bath set at 37°C, **C(g)**. Stir the contents at 170 rpm for 10 min to equilibrate to 37°C. Alternatively, transfer the bottles (without stirrer bar) to a Grant OLS 200 shaking incubation bath, **C(g)**, or similar, and secure in place with the shaker frame springs or a polypropylene holder, Figure **2107.16B**, and shake at 150 rpm in orbital motion for 10 min.
  3. *Incubation with PAA plus AMG.—*Add 5.0 mL PAA/AMG solution, **B(e)**, (PAA 4 KU/5 mL and AMG 1.7 KU/5 mL) to each bottle, cap the bottles and incubate the reaction solutions at 37°C with stirring at 170 rpm for for 4 h ± 5 min using a magnetic stirrer bar and a 2mag Mixdrive 15 magnetic stirrer apparatus; alternatively incubate in a shaking water bath maintained at 37°C at 150 rpm (orbital motion) for 4 h ± 5 min. Alternatively, if employing the ammonium sulphate suspension of PAA/AMG [PAA (2 KU/mL)/AMG (0.85 KU/mL); *see* **B(e)**, alternative], gently swirl the suspension before use and add 2.0 mL of this suspension and 3 mL maleate buffer, **B(l)**, or MES buffer, **B(m)**, to each bottle and incubate as indicated.
  4. *Adjustment of pH and inactivation of PAA and AMG.—*After 4 h of incubation, remove all sample bottles from the stirring or shaking water bath, and immediately add 3.0 mL 0.75 M Tris base solution, **B(n)**, to adjust the pH to approximately 8.2 (7.9–8.4), at which pH AMG has no activity. Immediately, slightly loosen the caps of the sample bottles, place the bottles in a boiling water bath (nonshaking; 95–100°C), and incubate for 20 min with occasional agitation (by hand). This inactivates both PAA and AMG. With a thermometer, ensure that the final temperature of the bottle contents is >90°C. Checking just one bottle is adequate. (At the same time, if only one shaker bath is available, increase the temperature of the shaking incubation bath to 60°C in readiness for the protease incubation step).
  5. *Cool.—*Remove all sample bottles from the hot water bath (use appropriate gloves) and cool to approximately 60°C.
  6. *Protease treatment.—*Suspend the protease, **B(f)**, by carefully swirling the bottle and add 0.1 mL protease suspension using a positive displacement dispenser (the solution is thick) to each bottle and incubate at 60°C for 30 min.
  7. *pH adjustment.—*Add 4.0 mL 2 M acetic acid, **B(o)**, to each bottle and mix. This gives a final pH of approximately 4.3.
  8. *Add internal standard.—*To each sample, add either 1 mL glycerol internal standard solution (100 mg/mL), **B(g),** or 1 mL of DEG internal standard solution (100 mg/mL), **B(h)**, or alternatively, add 1 mL of each.
  9. Proceed to step **G(a)** for determination of IDF.

**Table 2.** Comparison of AOAC method data

AOAC Method	Title	s_r_, g/100 g^a^	RSD_r_, %^b^	s_R_, g/100 g^c^	RSD_R_, %^d^
**985.29**	Total Dietary Fiber in Foods	0.15–0.99	0.56–66.25	0.27–1.36	1.58–66.25
**991.42**	Insoluble Dietary Fiber in Food and Food Products	0.41–2.82	0.86–10.38	0.62–9.49	3.68–19.44
**991.43**	Soluble, Insoluble and Total Dietary Fiber in Food and Food Products	0.36–1.06	1.50–6.62	0.41–1.43	1.58–12.17
**992.16**	Total Dietary Fiber	0.18–1.01	1.48–14.73	0.22–2.06	4.13–17.94
**993.19**	Soluble Dietary Fiber in Foods and Food Products	0.49–1.15	1.74–5.93	0.79–2.05	2.41–7.01
**994.13**	Total Dietary Fiber (Determined as Neutral Sugar Residues, Uronic Acid Residues and Klayson Lignin)	0.32–2.88	1.80–6.96	0.52–4.90	4.80–11.30
**2001.03**	Dietary Fiber Containing Supplemented Resistant Maltodextrins (RMD)	0.02–1.63	1.33–6.10	0.04–2.37	1.79–9.39
**2002.02**	Resistant Starch in Starch and Plant Products	0.08–2.66	1.97–4.12	0.21–3.87	4.48–10.90
**2009.01**	Total Dietary Fiber in Foods	0.41– 1.43	1.65–12.34	1.18–5.44	4.70–17.97
**2011.25**	Insoluble, Soluble and Total Dietary Fiber by an Enzymatic-Gravimetric Method and LC	0.47–1.41	2.43–8.60	0.95–3.14	6.85–14.48
**2017.16**	Total Dietary Fiber in Foods (Codex Definition) by a Rapid Enzymatic-Gravimetric Method and Liquid Chromatography	0.27–0.76	1.22–6.52	0.54–3.99	2.14–10.62
**2022.01^e^**	Insoluble, Soluble and Total Dietary Fiber (Codex Definition) in Foods by an Enzymatic-Gravimetric Method and Liquid Chromatography	0.59–1.35	1.58–3.57	1.11–3.05	4.55–9.26

a S_r_ = Within laboratory variability.

b RSD_r_ = Within laboratory relative variability.

c S_R_ = Between laboratory variability.

d RSD_R_ = Between laboratory relative variability.

e Values for total dietary fiber with DEG internal standard. Very similar values were obtained with glycerol internal standard (Table **2022.01C**).

### G. Determination of IDF

- *Filtration setup.—*Tare two crucibles containing Celite to nearest 0.1 mg for each sample. Wet and redistribute the bed of Celite in the crucible, using 15 mL 78% (v/v) EtOH (or IMS), **B(b)***,* from wash bottle. Apply suction to crucibles to draw Celite onto fritted glass as an even mat. Discard this filtrate.
- *Filtration*.—Using vacuum, filter enzyme digest, **F(b) (**9**)**, through crucible. With the aid of a wash bottle use 21 mL or less of deionized water (warmed to 60°C) to quantitatively transfer remaining particles to crucible and rinse residue. Retain combined filtrate and transfer water and set aside for determination of SDFP, **H(a)**.
- *Wash.—*Using vacuum, wash residue two times each with 15 mL portions of 78% ethanol, 95% ethanol, and acetone. *Note*: A delay in washing IDF residues with 78% ethanol, 95% ethanol, and acetone may cause inflated IDF values. Discard these washings.
- *Dry crucibles.—*Loosely cover the crucibles with aluminum foil to prevent sample loss, and then dry the crucibles containing residue overnight in a 103°±2°C oven.
- *Cool crucibles.—*Cool crucibles in desiccators for approximately 1 h. Weigh crucible containing IDF residue and Celite to nearest 0.1 mg. To obtain residue weight, subtract tare weight, i.e., weight of dried crucible and Celite.
- *Protein and ash determination.—*The residue from one crucible of the duplicate is analyzed for protein, and the residue of the second duplicate is analyzed for ash. Perform protein analysis on residue using Kjeldahl or combustion methods. (Exercise caution when using a combustion analyzer for protein in the residue. Celite volatilized from the sample can clog the transfer lines of the unit.) Use 6.25 factor for all cases to calculate mg of protein (the protein content in a foodstuff is estimated by multiplying the nitrogen content by a nitrogen-to-protein conversion factor, usually set at 6.25. This historical factor assumes the nitrogen content of proteins to be 16%). For ash analysis, incinerate the second residue for 5 h at 525°C. Cool in desiccator and weigh to nearest 0.1 mg. Subtract crucible and Celite tare weight to determine ash.
- Proceed to determination of SDF as SDFP, **H(a)**, and SDFS, **I(a).**

### H. Determination of SDFP

- *Precipitation.—*To each sample filtrate, add any remaining water from the IDF transfer and rinse, **G(b)**, or additional water if necessary to bring the total volume to ∼70 mL, then add 320 mL (measured at room temperature) of 95% (v/v) EtOH or IMS, **B(a)**, preheated to 60°C, and mix thoroughly. Allow the SDFP precipitate to form at room temperature for 60 min or overnight.
- *Filtration setup.—*Tare crucible containing Celite to nearest 0.1 mg. Wet and redistribute the bed of Celite in the crucible, using 15 mL of 78% (v/v) EtOH or IMS, **B(b)***,* from wash bottle. Apply suction to crucible to draw Celite onto fritted glass as an even mat. Discard this filtrate.
- *Filtration*.—Using vacuum, filter SDFP precipitate, **H(a)**, from supernatant through crucible. Using a wash bottle with 78% (v/v) EtOH or IMS, **B(b)**, quantitatively transfer all remaining particles to crucible. Retain filtrate and washings as **H(c).**
- *Wash.—*Using a vacuum, wash residue successively with two 15 mL portions each of 78% (v/v) EtOH or IMS, 95% (v/v) EtOH or IMS, and acetone. Add these washings to fraction **H(c)** and retain for determination of SDFS.
- *Dry crucibles.—*Loosely cover the crucibles with aluminum foil to prevent sample loss, and then dry the crucibles containing residue overnight in 105°C oven.
- *Cool crucibles.—*Cool crucibles in desiccators for approximately 1 h. Weigh crucible containing dietary fiber residue and Celite to nearest 0.1 mg. To obtain residue weight, subtract tare weight, i.e., weight of dried crucible and Celite.
- *Protein and ash determination.—*Residue from one crucible of the duplicate is analyzed for protein, and the residue of the second duplicate is analyzed for ash. Perform protein analysis on residue using Kjeldahl or combustion methods. (Exercise caution when using a combustion analyzer for protein in the residue. Celite volatilized from the sample can clog the transfer lines of the unit.) Use 6.25 factor for all cases to calculate mg of protein. For ash analysis, incinerate the second residue for 5 h at 525°C. Cool in desiccator and weigh to nearest 0.1 mg. Subtract crucible and Celite tare weight to determine ash.

### I. Determination of SDFS

Proper deionization of the filtrate is an essential part of obtaining quality chromatographic data on SDFS. *See*Figure **2022.01E** for patterns of glycerol and d-glucose in the presence and absence of buffer salts. To ensure that the resins being used are of adequate deionizing capacity, add 0.1 mL protease suspension, **B(f),** to 40 mL of either maleate buffer, **B(l),** or MES buffer, **B(m),** along with 3.0 mL 0.75 M Tris base solution, **B(n),** 4.0 mL 2M acetic acid, **B(o),** 1 mL glycerol internal standard (100 mg/mL), **B(g),** and 1 mL d-glucose solution (100 mg/mL).

**Figure 2022.01E.**
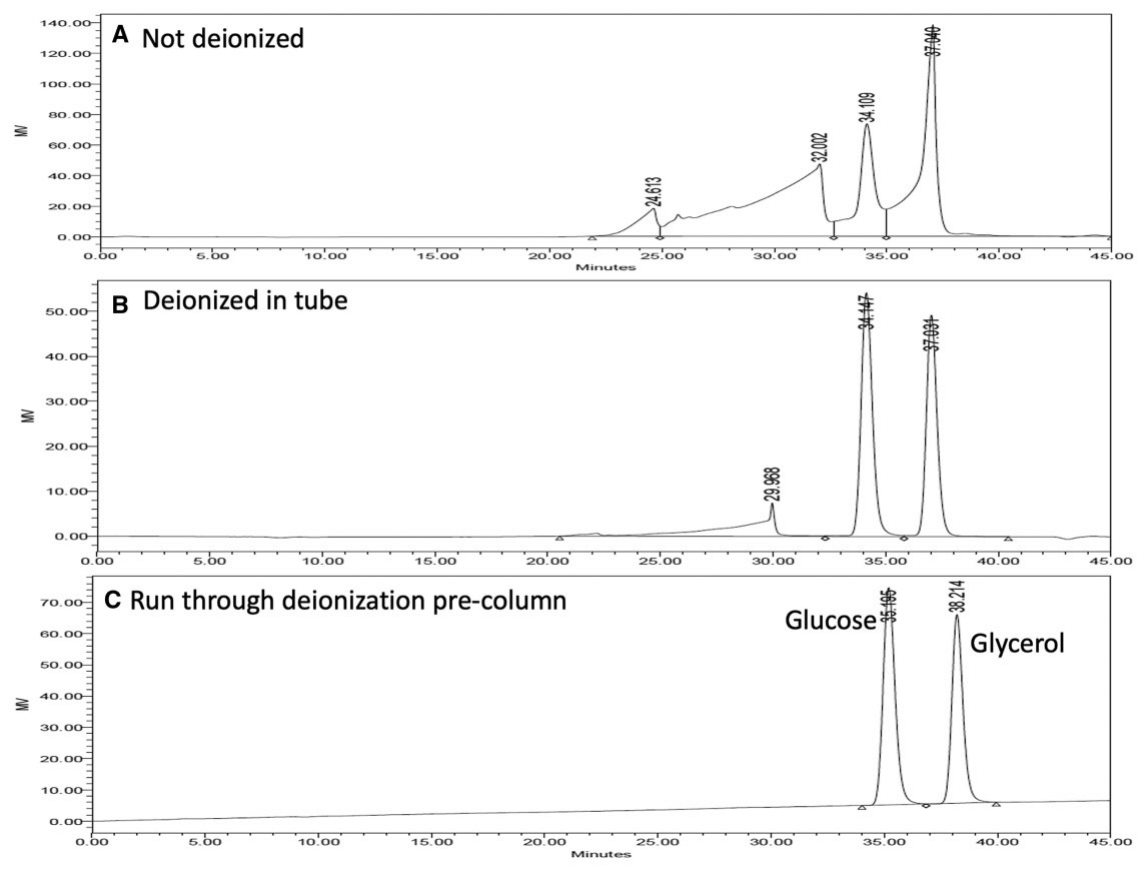
Chromatograms on TSKgel G2500PW_XL_ columns of glucose/glycerol mixtures. A mixture of glycerol (100 mg) and glucose (100 mg) was analyzed according to the RINTDF procedure. The ethanolic filtrate (for SDFS determination) was concentrated to dryness and redissolved in 32 mL deionized water. (a) A sample of this was analyzed by HPLC directly with no deionization and no Bio-Rad deionization pre-cartridges in place; (b) a sample (5 mL) was deionized by mixing with 1.5 g Amberlite FPA53 (OH^−^) and 1.5 g Ambersep 200 (H^+^) resins over 5 min and the supernatant was analyzed by HPLC with no Bio-Rad deionization pre-cartridges in place; and (c) a sample (b) was analyzed with a Bio-Rad deionization pre-cartridges in place. Deionization with resins in a polypropylene tube, as described here, removes >95% of the salt from the sample, thus ensuring more efficient use of the expensive Bio-Rad deionization pre-cartridges. This deionization step increases the effectiveness of the deionization cartridges and allows up to 10 times more samples to be chromatographed before the need to regenerate or replace the deionization cartridges.

Concentrate this solution to dryness on a rotary evaporator and re-dissolve the residue in 32 mL deionized water and store in 5 mL aliquots in 15 mL polypropylene tubes in a freezer for future use. To 5 mL of this solution in a 15 mL polypropylene tube, add 1.5 g Amberlite FPA53 (OH^−^) resin, **B(u),** and 1.5 g of Ambersep 200 (H^+^) resin, **B(u),** and swirl the contents regularly over 5 min (Figure **2022.01F**). Allow the resin to settle and remove the supernatant solution (∼2 mL) with a syringe, **C(cc),** and filter through a 0.45 μm membrane, **C(z).** Inject an aliquot (50 µL) of this solution onto the TSKgel G2500PW_XL_ columns [Bio-Rad deionization pre-cartridges, **C(v),** in place; Figure **2022.01G**]. No salt peaks should be seen on HPLC. Retain this solution at −20°C for periodic evaluation of the ability of the Bio-Rad deionization cartridges to completely remove all salt remaining in the partially desalted sample.

**Figure 2022.01F.**
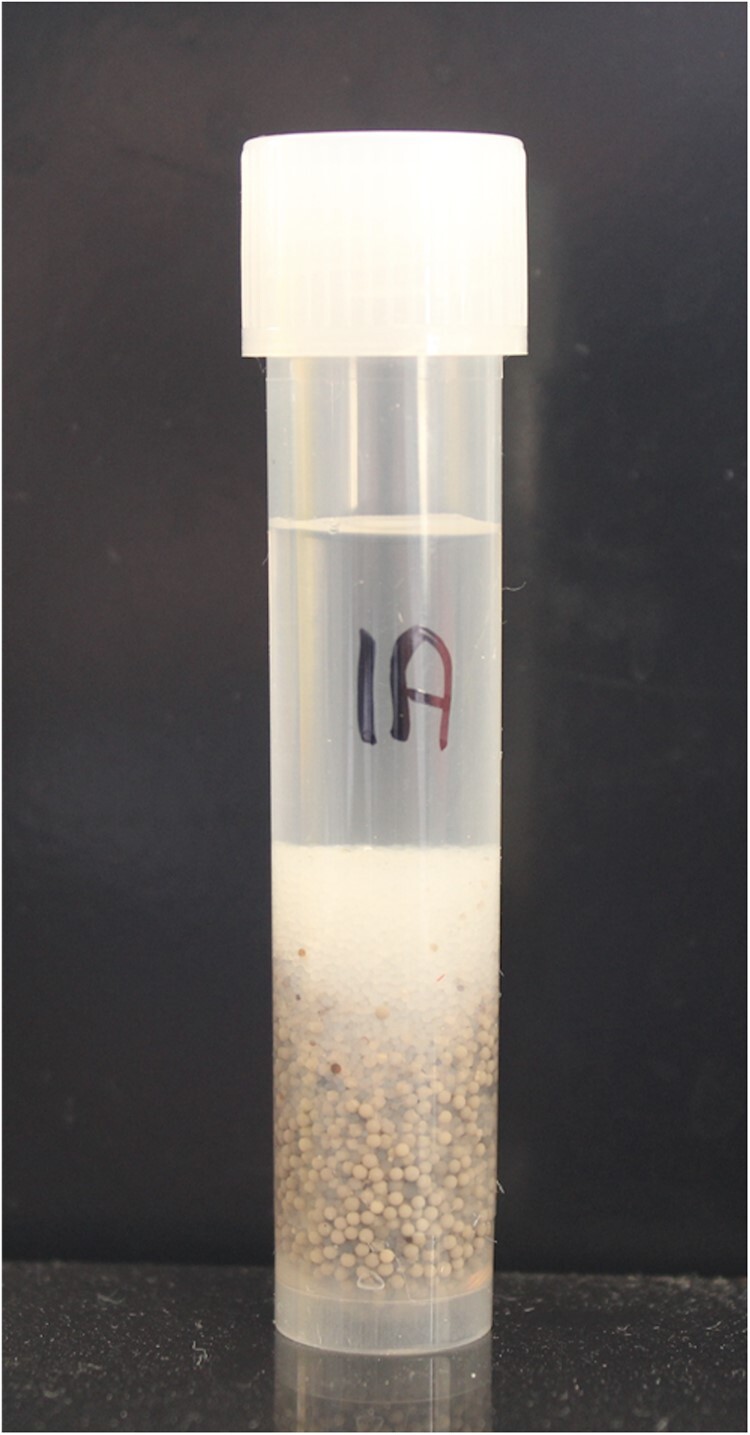
Deionization of samples for HPLC. Five milliliters concentrated eluate mixed with 1.5 g Amberlite FPA53 (OH^−^) and 1.5 g Ambersep 200 (H^+^) resins in a polypropylene tube. This treatment removes 90–95% of the salt in the sample which extends the effective life of the Bio-Rad deashing cartridges B(v) by > 10-fold.

- *Filtrate recovery, deionization, and LC analysis.—*Set aside the filtrate plus washings from one of the sample duplicates, **H(c),** to use in case of spills or if duplicate SDFS data are desired. Transfer 25% (approximately 130 mL) of the filtrate plus washings, **H(c),** of the other duplicate into a 500 mL evaporator flask and concentrate with a rotary evaporator to dryness at 50°C. (*Note:* It is not essential to quantitatively transfer all solution because SDFS is determined by the ratio of these peaks on HPLC to that of either the glycerol or DEG internal standard.)
- *Deionization of sample.—*Dissolve the residue in the evaporator flask in 8 mL deionized water and transfer most of this solution to a 15 mL polypropylene container, **C(s)**. Transfer 5 mL of this solution to a 15 mL polypropylene tube, **C(s),** containing 1.5 g Amberlite FPA53 (OH^−^) resin and 1.5 g Ambersep 200 (H^+^; Figure **2022.01F**). Cap the container and invert the contents regularly over 5 min. Alternatively, if the ammonium sulphate suspension of PAA/AMG is used for starch digestion [*see* **B(e),** alternative], then use 2 g Amberlite FPA53 (OH^−^) resin and 2 g Ambersep 200 (H^+^) to ensure effective removal of most of the ions in the sample.
- *Prepare samples for LC analysis.—*Remove a sample (approximately 1.5–2.0 mL) of the supernatant solution from the resin slurry (Figure **2017.16F**) with a syringe, **C(cc),** and filter through a 0.45 μm membrane, **C(z)**. Use this solution as the sample extract for step **I(f)**. HPLC patterns for non-deionized sample, sample deionized with resin in tube, and sample of preparation desalted on TSKgel G2500PW_XL_ columns through Bio-Rad deionization pre-cartridges are shown in Figure **2022.01G**.
- *Determine the response factor for d-glucose.—*Because d-glucose provides an LC RI response equivalent to the response factor for the nondigestible oligosaccharides that make up SDFS, d-glucose is used to calibrate the LC and the response factor is used for determining the mass of SDFS. Use a 100 μL LC syringe, **C(dd)**, to fill the 50 μL injection loop with either the d-glucose/glycerol internal standard solution **B(j)**, or the d-glucose/DEG internal standard solution, **B(k)**. Inject in duplicate. Calculate the response factor according to **L(a)**.
- *Calibrate the area of the chromatogram to be measured for SDFS.—*Use a 100 μL LC syringe, **C(dd),** to fill the 50 μL injection loop with retention time standard, **C(i)**. Inject in duplicate. Determine the demarcation point between degree of polymerisation (DP) 2 and DP 3 oligosaccharides (disaccharide maltose versus higher oligosaccharides; Figure **2022.01B**).
- *Determine peak areas of SDFS (PA_SDFS_) and internal standard (PA_IS_) in chromatograms of sample extracts.—*Inject sample extracts, **I(c)**, on the LC system. Record areas of all peaks with DP greater than the DP 2/DP 3 demarcation point (obtained for the retention time standard) as PA_SDFS_. Record the peak area of internal standard as PA_IS_ (either glycerol or DEG).
- Proceed to step **L(b)**.

**Figure 2022.01G.**
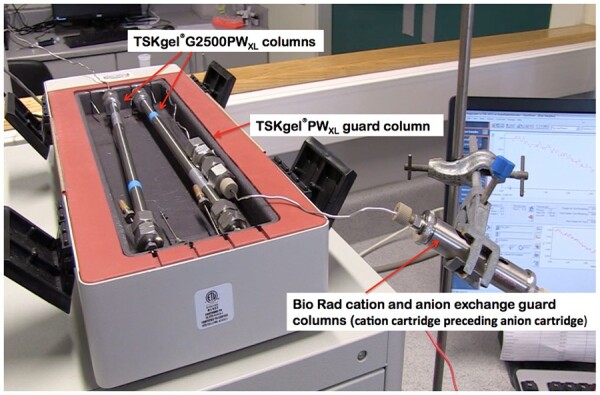
HPLC setup for measurement of SDFS, highlighting the two TSKgel G2500PW_XL_ columns, TSKgel PW_XL_ guard column in a column heater module, and a Bio-Rad cartridge containing cation and anion deionization cartridges.

*Note*: For samples containing significant levels of lactose or isomaltose, clear delineation of SDFS from disaccharides on chromatography on TSKgel G2500PW_XL_ columns is difficult. This problem can be resolved by hydrolyzing lactose and/or isomaltose to glucose and galactose as follows: To a 5 mL aliquot of the SDFS fraction add 1 mL 1 M sodium acetate buffer (pH 4.0) to give a final pH of ∼4.5. Then add 0.1 mL of a suspension of β-galactosidase (EC 3.2.1.23; 2000 U/mL) and oligo-α-1,6-glucosidase (EC 3.2.1.10; 2000 U/mL, Megazyme Cat. No. E-BGOG) and incubate at 40°C for 30 min. Heat the solution in a boiling water bath for 3 min, cool to room temperature, and deionize by adding add 1.5 g of both Amberlite FPA53 (OH^−^) and Ambersep 200 (H^+^) resins and mixing over 5 min. Filter solutions through a 47 mm, 0.45 µm syringe filter and apply to TSKgel G2500PW_XL_ HPLC columns with in-line deionization. Under these conditions essentially all of the galacto-oligosaccharides (GOS) in the sample will also be hydrolyzed to galactose and glucose. Hydrolysis of lactose and isomaltose by a mixture of oligo-α-1,6-glucosidase and β-galactosidase (E-BGOG) is shown in Figure **2022.01H**.

**Figure 2022.01H.**
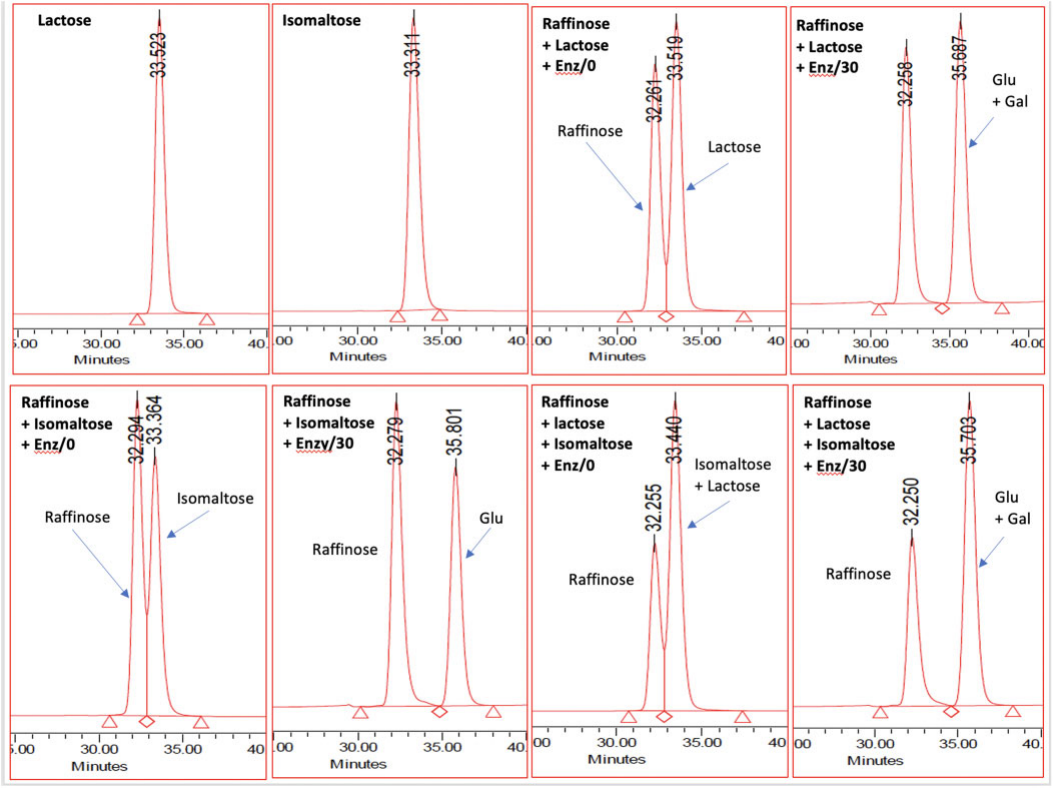
Chromatography of lactose, isomaltose, and various mixtures of raffinose, lactose and isomaltose on two TSKgel G2500PW_XL_ columns in series. Solvent: distilled water; flow rate: 0.5 mL/min; and temperature 80°C. Mixtures of raffinose (approximately 5 mg/mL) and lactose (approximately 5 mg/mL) and/or isomaltose (5 mg/mL) are chromatographed before and after incubation with 0.1 mL of a suspension of β-galactosidase (EC 3.2.1.23; 2000 U/mL) and oligo-α-1,6-glucosidase (EC 3.2.1.10; 2000 U/mL, Megazyme Cat. No. E-BGOG) plus 0.1 mL 0.2 M sodium acetate buffer (pH 4.5) at 40°C for 30 min.

### J. Calculation of IDF (by Gravimetry)

#### (a) Blank (B, mg) determination


$$B\;\left(\text{mg}\right)=\frac{{\text{BR}}_{1}+{\;\text{BR}}_{2\;\;\;}}{2}-P_{B}-{\;A}_{B}$$

where BR_1_ and BR_2_ = residue mass, in mg, for duplicate IDF blank determinations, respectively; and P_B_ and A_B_ = mass, in mg, of protein and ash, respectively, determined on first and second blank residues.

#### (b) IDF determination


$$\begin{array}{c} \text{IDF}\;\left(\text{mg}/100g\right)=\frac{\left[\left(R_{1}+{\;R}_{2}\right)/2\;–\;P\;–\;A\;–\;B\right]}{\left(M_{1}+{\;M}_{2}\right)/2}\times 100\\ \text{IDF}\;\left(g/100g\right)=\frac{\text{IDF}\;\left(\text{mg}/100g\right)}{1000} \end{array}$$

where R_1_ = IDF residue mass 1 from M_1_ in mg; R_2_ = IDF residue mass 2 from M_2_ in mg; M_1_ = test portion mass 1 in g; M_2_ = test portion mass 2 in g; P = protein mass in mg from R_1_; A = ash mass in mg from R_2_; and B = IDF blank from **J(a)**.

### K. Calculation of SDFP (by Gravimetry)

#### (a) Blank (B, mg) determination


$$B\;\left(\text{mg}\right)=\frac{{\text{BR}}_{1}+{\;\text{BR}}_{2\;\;\;}}{2}-P_{B}-{\;A}_{B}$$

where BR_1_ and BR_2_ = residue mass, in mg, for duplicate SDFP blank determinations, respectively; and P_B_ and A_B_ = mass, in mg, of protein and ash, respectively, determined on the first and second blank residues.

#### (b) SDFP determination


$$\begin{array}{c} \text{SDFP}\;\left(\text{mg}/100g\right)=\frac{\left[\left(R_{1}+{\;R}_{2}\right)/2\;–\;P\;–\;A\;–\;B\right]\;}{\left(M_{1}+{\;M}_{2}\right)/2}\times 100\\ \text{SDFP}\;\left(g/100g\right)=\frac{\text{SDFP}\;\left(\text{mg}/100g\right)}{1000} \end{array}$$

where R_1_ = SDFP residue mass 1 from M_1_ in mg; R_2_ = SDFP residue mass 2 from M_2_ in mg; M_1_ = test portion mass 1 in g; M_2_ = test portion mass 2 in g; P = protein mass, in mg, from R_1_; A= ash mass, in mg, from R_2_; and B = SDFP blank from **K(a)**.

### L. Calculation of SDFS (by HPLC)

- *Determination of d-glucose response factor*.—Obtain the values for the peak areas of d-glucose and internal standard (glycerol or DEG) from duplicate chromatograms. The ratio of peak areas of d-glucose/internal standard (glycerol or DEG) to the ratio of masses of d-glucose/internal standard (glycerol or DEG) is the “response factor.” The average response factor for glycerol/d-glucose is approximately 0.82, whereas that for DEG/d-glucose is approximately 0.75.
  $$\text{Response}\;\text{factor}\;\left(\text{Rf}\right)=\frac{{\text{PA}}_{\text{IS}}}{{\text{PA}}_{\text{Glu}}}\times \frac{{\text{Wt}}_{\text{Glu}}}{{\text{Wt}}_{\text{IS}}}$$

where PA_Glu_ = peak area of d-glucose; PA_IS_ = peak area of glycerol or DEG internal standard; Wt_Glu_ = mass of d-glucose/mL in standard, **B(j)** and Wt_IS_ = mass of glycerol/mL **B(j);** or Wt_Glu_ = mass of d-glucose/mL in standard, **B(k)** and Wt_IS_ = mass of DEG/mL in standard **B(k)**.

- *SDFS determination.—*
$$\begin{array}{c} \text{SDFS}\;\left(\text{mg}/100g\right)=\frac{\text{Rf}\;\times \;{\text{Wt}}_{\text{IS}}\;\times \;{\text{PA}}_{\text{SDFS}\;\;}}{{\text{PA}}_{\text{IS}}}\;{\times \;}_{}\frac{100}{M}\\ \text{SDFS}\;\left(g/100g\right)=\frac{\text{SDFS}\;\left(\text{mg}/100g\right)}{1000} \end{array}$$

where Rf = response factor from **L(a)**; Wt_IS_ = mass in mg of internal standard contained in 1 mL internal standard solution (100 mg), **B(g) or B(h)**, pipetted into sample before filtration; PA_SDFS_ = peak area of the SDFS; PA_IS_ = peak area of the internal standard; and M = test portion mass (g), M_1_ or M_2_, of the sample whose filtrate was concentrated and analyzed by LC.

### M. Calculation of SDF


SDF (g/100g)=SDFP [g/100 g;K(b)]+SDFS [g/100 g;L(b)]

### N. Calculation of TDF


TDF (g/100g)=IDF [g/100g;J(b)]+SDF (g/100g;M)

## Results and Discussion

Samples were selected to cover a wide range of foods, feeds, and ingredients for which insoluble, soluble, and TDF values would be useful. The samples chosen contained various levels of IDF, SDFP, and SDFS to ensure accurate measurement of all dietary fiber components. All materials were prepared, dried, dispensed into sealed tubes, and blind coded before dispatch to avoid possible deterioration during shipping.

### Practice Sample Testing

To ensure adequate method performance and a good understanding of the method by collaborators, practice samples were analyzed by all the participating laboratories. Collaborators were sent four samples, labelled P1–P4, along with copies of the method, an Excel-based data calculator, and the required enzymes, control solutions, and ion exchange resins. Each laboratory was asked to analyze a single test portion of each sample, to ask questions regarding the procedures, and to provide feedback to the method author. The results of the analysis on the practice samples are shown in Table 1. In the gravimetric determinations of IDF and SDFP, no specific problems were identified by the collaborators. Some problems and misunderstandings occurred in the measurement of the SDFS fraction by HPLC. The need for use of the stated HPLC columns, with ongoing maintenance, and correct deionization of samples using resin in tubes together with in-line deionization was again reiterated. The importance of maintaining the sample in suspension during the incubation with PAA/AMG was again highlighted.

The results of the practice samples were typical for dietary fiber methods. Repeatability values were within the range of performance limits typically found for dietary fiber methods, wherein a significant number of manual steps are necessary to perform the assay. Samples were analyzed for IDF and SDFP gravimetrically and SDFS by HPLC. The reproducibility SD (S_R_) for TDF ranged from 0.79 to 1.91 g/100 g, and the RSD_R_ ranged from 6.69 to 11.68%, values consistent with those reported for analyses of similar samples with other dietary fiber assay formats (Table 2). A similar range of RSD_R_ values were obtained for IDF i.e., 5.16 to 13.45%. The higher RSD_R_ values for the SDF fraction are in line with a similar range obtained in other studies (AOAC **2011.25**) and were thought to be due mainly to the unfamiliarity of collaborators with the requirements of this assay.

It was concluded that the method was ready for a collaborative study. It is generally known that some food samples contain glycerol either as a natural component or as an added ingredient, thus glycerol is not an ideal internal standard for use in SDFS determinations by HPLC. Some laboratories routinely use DEG as internal standard as it elutes as a discrete peak separate from all other components in the samples. To gain further insight into the relative value of these two compounds as internal standards, collaborators were requested to employ both compounds in the MLV study.

### Collaborative Study Results

All of the 17 laboratories that analyzed the practice samples completed the study and 16 reported a full set of results with the glycerol internal standard; 14 laboratories reported a full set of results with the DEG internal standard; and a fifteenth laboratory reported a partial set of results with the DEG internal standard. Collaborating laboratory data were evaluated statistically according to AOAC guidelines using an AOAC workbook (version 4.8).

In measurement of IDF with the glycerol internal standard, of the eight valid pairs of assay results reported from 16 collaborators, Laboratories 1, 5, and 8 reported a single duplicate pair outlier and Laboratories 9 and 11 reported two duplicate pairs of outliers (*see*Table 3a). With the DEG internal standard employed, Laboratories 5 and 8 reported a single duplicate pair outlier and Laboratories 9 and 11 reported two duplicate pairs of outliers (*see*Table 4a). For SDF with glycerol internal standard (IS), **B(g)**, Laboratory 5 reported a single statistical outlier pair (samples K and N) and Laboratories 4 and 9 reported two statistical outliers (*see*Table 3b); with DEG IS, single outliers were reported by Laboratories 9 and 13. For TDF with glycerol IS, Laboratories 4, 9, 11, and 12 reported single outliers (*see*Table 3c); with DEG IS, single outliers were reported for Laboratories 9, 11 and 13 (*see*Table 4c).

**Table 3.** Interlaboratory study results using glycerol internal standard

Sample^a^																
Lab	A & F		B & J		C & M		D & L		E & P		G & H		I & O		K & N	
(a) Determined insoluble dietary fiber (g/100 g as is)																
1	19.90	21.29	11.93	12.03	9.08^b^	9.90^b^	12.54	13.27	12.89	12.33	24.54	20.86	8.75	7.12	11.75	11.88
2	NR^c^	NR	NR	NR	NR	NR	NR	NR	NR	NR	NR	NR	NR	NR	NR	NR
3	19.63	20.18	12.64	12.86	8.78	8.82	13.05	14.11	11.27	12.36	23.52	24.84	5.60	5.59	12.06	12.58
4	21.34	19.61	12.57	12.54	7.03	8.74	13.30	13.45	11.11	12.18	23.23	23.81	8.22	8.56	12.00	11.89
5	17.69	18.09	11.93	11.82	6.21	5.54	13.06	12.06	10.47	10.76	16.94^d^	22.57^d^	3.82	4.39	11.55	11.55
6	18.79	17.29	11.74	12.67	6.52	6.47	14.60	14.54	12.31	12.67	22.00	22.39	3.69	4.23	11.82	11.98
7	19.55	18.82	12.49	12.89	6.78	6.84	15.23	13.57	12.29	11.45	23.65	22.69	5.19	4.35	12.22	11.87
8	19.74	19.51	13.25	13.56	10.19^b^	10.47^b^	13.04	12.57	14.77	15.49	26.21	26.03	8.61	8.74	12.13	12.71
9	17.83	19.43	12.00	11.35	5.74^b^	3.88^b^	12.44	11.34	10.79	9.64	21.54	19.83	3.97	4.04	10.63^d^	11.69^d^
10	22.24	19.47	12.60	12.99	7.42	8.29	13.39	13.34	10.80	11.16	23.10	23.43	4.08	4.29	12.09	12.17
11	22.48	23.14	12.45	11.37	6.19	6.59	16.78^d^	21.19^d^	17.02^b^	15.59^b^	21.89	22.10	3.03	4.22	12.77	12.79
12	20.50	20.57	14.09	14.02	8.58	7.59	15.99	14.32	14.44	14.47	23.61	24.51	6.30	7.08	11.58	10.99
13	20.45	22.27	13.11	12.68	7.34	8.47	13.74	13.79	12.65	12.79	23.06	23.42	4.94	7.63	12.11	11.90
14	19.39	19.13	12.98	12.89	9.12	8.50	12.49	12.61	13.88	11.88	23.12	23.74	8.04	8.15	11.78	11.77
15	21.10	20.31	13.48	13.38	7.03	6.57	12.68	14.03	14.19	14.19	22.83	24.11	5.12	7.08	11.96	12.18
16	19.25	19.82	12.56	13.37	6.63	6.21	12.85	12.69	11.37	11.32	22.05	21.10	4.95	3.80	11.89	11.97
17	18.81	19.36	11.80	11.62	6.61	6.43	12.86	12.86	11.53	11.74	21.45	21.33	3.45	3.40	11.64	11.58

(b) Determined soluble dietary fiber (g/100 g as is)																
1	6.53	6.26	9.34	9.39	23.91	22.71	9.85	9.31	9.30	9.30	18.37	18.92	31.29	32.88	13.44	13.56
2	NR^b^	NR	NR	NR	NR	NR	NR	NR	NR	NR	NR	NR	NR	NR	NR	NR
3	6.92	6.68	9.67	9.30	23.43	21.35	9.88	9.86	10.16	9.44	19.41	18.53	31.57	31.81	13.41	13.98
4	6.54	6.77	9.63^c^	13.01^c^	25.31	28.45	9.88	10.08	9.61	10.17	17.37	17.25	36.94^d^	42.48^d^	15.98	16.74
5	7.54	7.35	10.17	11.00	26.17	25.78	10.80	10.51	11.42	10.96	21.71	19.96	36.97	37.46	18.61^d^	18.30^d^
6	7.51	8.39	9.73	9.08	24.15	23.21	11.92	10.10	12.21	11.16	17.20	16.91	32.82	30.83	12.53	11.58
7	6.63	6.77	9.29	9.44	22.99	21.96	9.90	11.00	9.88	10.00	17.85	17.84	30.63	29.84	13.71	13.54
8	7.37	6.77	10.46	10.50	23.94	23.32	10.92	11.19	11.89	12.01	19.01	18.63	29.19	29.97	16.38	16.31
9	8.11	8.85	11.99	11.75	28.88	29.97	11.78	10.67	11.70	11.29	22.59	23.58	55.16^d^	51.08^d^	22.83^d^	22.25^d^
10	6.31	7.35	9.12	8.90	20.80	20.81	10.31	10.23	10.42	9.65	15.92	16.18	30.22	30.27	12.01	11.66
11	8.26	8.05	11.87	10.99	26.71	24.93	12.36	11.54	12.05	13.52	21.82	21.85	34.51	34.00	14.40	14.69
12	8.19	7.40	9.73	9.95	22.63	20.31	8.80	10.07	10.55	10.56	16.98	16.99	37.68	40.36	13.07	13.86
13	8.74	8.72	10.87	10.62	27.51	21.65	11.17	10.80	10.80	12.07	16.01	15.75	36.93	38.36	10.05	10.02
14	7.41	7.14	10.65	10.00	22.17	21.92	10.63	10.50	11.45	10.59	16.59	16.64	32.17	32.14	14.00	14.20
15	8.30	9.02	9.90	10.84	22.61	21.07	12.17	11.94	10.94	12.65	16.28	16.23	37.05	34.90	10.85	10.65
16	6.99	7.17	9.64	10.23	21.83	22.05	10.33	9.93	10.29	9.83	17.31	17.50	33.26	32.06	13.18	13.36
17	6.59	6.37	9.08	9.00	25.57	26.09	9.49	9.07	9.84	9.90	18.04	16.88	30.83	30.92	13.47	14.35

(c) Determined total dietary fiber (g/100 g as is)																
1	26.44	27.56	21.27	21.42	32.98	32.61	22.39	22.57	22.18	21.63	42.91	39.78	40.04	39.99	25.19	25.44
2	NR^b^	NR	NR	NR	NR	NR	NR	NR	NR	NR	NR	NR	NR	NR	NR	NR
3	26.55	26.86	22.31	22.16	32.21	30.16	22.93	23.98	21.44	21.80	42.92	43.37	37.17	37.40	25.46	26.55
4	27.88	26.38	22.20^c^	25.54^c^	32.34	37.18	23.17	23.53	20.72	22.35	40.60	41.06	45.16	51.04	27.97	28.64
5	25.23	25.45	22.10	22.82	32.38	31.32	23.85	22.57	21.89	21.72	38.64	42.53	40.79	41.05	30.16	29.85
6	26.30	25.68	21.47	21.76	30.67	29.68	26.52	24.63	24.52	23.84	39.20	39.30	36.51	35.06	24.35	23.57
7	26.19	25.59	21.78	22.32	29.77	28.80	25.13	24.56	22.16	21.45	41.51	40.53	35.82	34.19	25.92	25.41
8	27.12	26.28	23.72	24.06	34.13	33.78	23.96	23.76	26.66	27.50	45.22	44.65	37.81	38.70	28.52	29.03
9	25.94	28.28	23.99	23.11	34.62	33.85	24.22	22.01	22.49	20.93	44.13	43.41	59.13^d^	55.13^d^	33.46	33.94
10	28.55	26.81	21.72	21.89	28.22	29.09	23.70	23.57	21.22	20.81	39.02	39.61	34.30	34.56	24.10	23.83
11	26.91	27.13	22.41	20.52	30.62	29.13	28.28^d^	31.14^d^	24.80	25.12	41.95	42.00	36.50	37.17	27.02	27.37
12	28.69	27.96	23.82	23.98	31.21	32.39	24.80	24.39	24.99	25.03	40.59	41.49	43.97	47.43	24.65^c^	26.77^c^
13	29.19	30.99	23.98	23.31	34.85	30.12	24.90	24.59	23.45	24.86	38.08	39.17	41.86	46.00	22.16	21.93
14	26.83	26.27	23.76	22.89	31.49	30.43	23.13	23.11	25.41	22.47	39.71	40.38	40.21	40.29	25.78	25.97
15	29.39	29.33	23.38	24.22	29.63	27.64	24.86	25.96	25.13	26.84	39.12	40.34	42.17	41.98	22.81	22.83
16	26.23	26.99	22.19	23.60	28.45	28.26	23.19	22.62	21.66	21.15	39.36	38.61	38.21	35.85	25.08	25.33
17	25.40	25.73	20.88	20.62	32.18	32.53	22.35	21.93	21.51	21.64	39.49	38.21	34.28	34.32	25.11	25.93

a A & F = Kidney beans; B & J = Rye crispbread; C & M = Chocolate; D & L = Carrots. E & P = Oat bran; G & H = Barley MAX flour; I & O = Miso soup powder; K & N = Nutrition bar (Fiber 1).

b Grubbs outlier.

c NR = Not reported.

d Cochran outlier.

**Table 4.** Interlaboratory study results using DEG internal standard

Sample^a^																
Lab	A & F		B & J		C & M		D & L		E & P		G & H		I & O		K & N	
(a) Determined insoluble dietary fiber (g/100 g as is)																
1	NR^b^	NR	NR	NR	NR	NR	NR	NR	NR	NR	NR	NR	NR	NR	NR	NR
2	19.67	19.75	12.87	11.87	6.36	6.39	13.07	13.39	11.30	11.82	22.16	23.60	4.20	5.07	11.69	11.74
3	NR	NR	NR	NR	NR	NR	NR	NR	NR	NR	NR	NR	NR	NR	NR	NR
4	21.34	19.61	12.57	12.54	7.03	8.74	13.30	13.45	11.11	12.18	23.23	23.81	8.22	8.56	12.00	11.89
5	17.69	18.09	11.93	11.82	6.21	5.54	13.06	12.06	10.47	10.76	16.94^c^	22.57^c^	3.82	4.39	11.55	11.55
6	18.79	17.29	11.74	12.67	6.52	6.47	14.60	14.54	12.31	12.67	22.00	22.39	3.69	4.23	11.82	11.98
7	NR	NR	NR	12.89	6.78	6.84	NR	13.57	NR	11.45	NR	NR	NR	4.34	12.22	11.87
8	19.74	19.51	13.25	13.56	10.19^d^	10.47^d^	13.04	12.57	14.77	15.49	26.21	26.03	8.61	8.74	12.13	12.71
9	17.83	19.43	12.00	11.35	5.74^d^	3.88^d^	12.44	11.34	10.79	9.64	21.54	19.83	3.97	4.04	10.63^c^	11.69^c^
10	22.24	19.47	12.60	12.99	7.42	8.29	13.39	13.34	10.80	11.16	23.10	23.43	4.08	4.29	12.09	12.17
11	22.48	23.14	12.45	11.37	6.19	6.59	16.78^c^	21.19^c^	17.02^d^	15.59^d^	21.89	22.10	3.03	4.22	12.77	12.79
12	20.50	20.57	14.09	14.02	8.58	7.59	15.99	14.32	14.44	14.47	23.61	24.51	6.30	7.08	11.58	10.99
13	20.45	22.27	13.11	12.68	7.34	8.47	13.74	13.79	12.65	12.79	23.06	23.42	4.94	7.63	12.11	11.90
14	19.39	19.13	12.98	12.89	9.12	8.50	12.49	12.61	13.88	11.88	23.12	23.74	8.04	8.15	11.78	11.77
15	21.10	20.31	13.48	13.38	7.03	6.57	12.68	14.03	14.19	14.19	22.83	24.11	5.12	7.08	11.96	12.18
16	19.25	19.82	12.56	13.37	6.63	6.21	12.85	12.69	11.37	11.32	22.05	21.10	4.95	3.80	11.89	11.97
17	18.81	19.36	11.80	11.62	6.61	6.43	12.86	12.86	11.53	11.74	21.45	21.33	3.45	3.40	11.64	11.58

(b) Determined soluble dietary fiber (g/100 g as is)																
1	NR^b^	NR	NR	NR	NR	NR	NR	NR	NR	NR	NR	NR	NR	NR	NR	NR
2	7.11	7.12	9.51	9.74	22.25	22.19	10.75	11.33	9.27	9.06	18.58	18.40	32.71	33.08	23.34	23.02
3	NR	NR	NR	NR	NR	NR	NR	NR	NR	NR	NR	NR	NR	NR	NR	NR
4	6.63	6.88	9.95	9.52	26.61	26.48	9.91	10.13	9.71	10.18	18.46	17.93	34.97	34.29	25.89	25.00
5	7.63	7.43	10.33	11.30	27.29	26.71	10.81	10.54	11.49	11.02	22.50	20.38	38.87	38.68	27.52	26.99
6	7.60	8.48	9.92	9.31	24.96	24.06	11.96	10.12	12.33	11.25	17.85	15.95	33.87	32.11	23.00	23.59
7	NR	NR	NR	10.40	23.71	23.41	NR	11.01	NR	10.21	NR	NR	NR	33.36	25.79	25.49
8	7.50	6.82	10.68	10.57	24.22	23.36	10.94	11.21	11.97	12.10	19.94	18.93	31.49	30.79	28.12	27.93
9	8.07	8.72	11.87	11.53	28.12	29.67	11.74	10.65	11.69	11.29	22.01	22.62	54.12^c^	50.67^c^	31.63	30.30
10	6.43	7.54	9.50	9.29	21.82	21.74	10.34	10.28	10.53	9.77	17.30	17.33	33.07	32.87	22.02	21.68
11	8.29	8.08	11.90	11.08	26.75	24.96	12.38	11.55	12.12	13.60	22.04	22.38	35.69	35.13	26.10	26.34
12	8.25	7.57	9.85	10.00	23.49	24.50	8.82	10.08	10.58	10.74	17.29	17.26	38.24	41.35	23.44	26.06
13	9.37	8.39	10.44	11.34	25.94	25.61	11.16	10.79	10.79	12.11	15.19	16.44	37.41	38.57	19.46^d^	16.30^d^
14	7.44	7.18	10.77	10.10	22.38	22.17	10.64	10.52	11.53	10.65	16.94	17.16	33.59	33.78	25.60	25.65
15	9.73	9.76	10.10	10.78	22.45	21.98	12.22	11.93	11.30	12.70	15.61	15.89	36.45	37.00	18.37	18.57
16	7.24	7.36	10.41	11.14	23.38	22.88	10.36	9.96	10.39	9.93	18.27	19.35	35.36	34.01	24.80	24.88
17	6.79	6.63	9.60	9.46	27.44	27.47	9.54	9.12	9.84	10.03	18.04	16.88	33.67	33.04	26.37	28.04

(c) Determined total dietary fiber (g/100 g as is)																
1	NR^b^	NR	NR	NR	NR	NR	NR	NR	NR	NR	NR	NR	NR	NR	NR	NR
2	26.78	26.87	22.38	21.61	28.61	28.58	23.82	24.72	20.57	20.88	40.74	42.00	36.91	38.15	35.03	34.76
3	NR	NR	NR	NR	NR	NR	NR	NR	NR	NR	NR	NR	NR	NR	NR	NR
4	27.97	26.48	22.52	22.05	33.64	35.21	23.20	23.57	20.82	22.37	41.69	41.74	43.19	42.85	37.88	36.89
5	25.32	25.52	22.26	23.12	33.50	32.25	23.87	22.60	21.95	21.79	39.43	42.95	42.69	43.07	39.07	38.53
6	26.39	25.77	21.66	21.98	31.48	30.52	26.56	24.66	24.63	23.93	39.86	38.34	37.56	36.34	34.81	35.57
7	NR	NR	NR	23.29	30.49	30.26	NR	24.58	NR	21.67	NR	NR	NR	37.72	38.01	37.36
8	27.24	26.33	23.94	24.13	34.41	33.83	23.98	23.78	26.74	27.59	46.15	44.96	40.10	39.53	40.25	40.64
9	25.90	28.15	23.87	22.88	33.86	33.54	24.18	21.98	22.49	20.93	43.55	42.45	58.09^c^	54.72^c^	42.26	41.99
10	28.67	27.00	22.10	22.28	29.24	30.02	23.73	23.61	21.33	20.93	40.39	40.76	37.15	37.16	34.11	33.85
11	26.94	27.15	22.44	20.62	30.66	29.15	28.30^c^	31.15^c^	24.87	25.19	42.17	42.52	37.68	38.30	38.72	39.02
12	28.75	28.13	23.93	24.02	32.07	32.09	24.81	24.40	25.02	25.21	40.89	41.76	44.54	48.43	35.03	37.05
13	29.82	30.66	23.55	24.02	33.28	34.08	24.90	24.58	23.44	24.91	38.26	39.86	42.35	46.20	31.57^d^	28.20^d^
14	26.83	26.31	23.76	23.00	31.49	30.67	23.13	23.12	25.41	22.53	40.06	40.90	41.62	41.93	37.39	37.43
15	30.83	30.07	23.58	24.16	29.51	28.55	24.90	25.96	25.49	26.88	38.44	40.00	41.57	44.08	30.33	30.75
16	26.49	27.18	22.96	24.51	30.01	29.08	23.22	22.65	21.76	21.25	40.32	40.45	40.32	37.81	36.70	36.85
17	25.61	25.99	21.40	21.08	34.05	33.90	22.41	21.98	21.51	21.77	41.18	39.50	37.12	36.44	38.01	39.62

a A & F = Kidney beans; B & J = Rye crispbread; C & M = Chocolate; D & L = Carrots. E & P = Oat bran; G & H = Barley MAX flour; I & O = Miso soup powder; K & N = Nutrition bar (Fiber 1).

b NR = Not reported.

c Cochran outlier.

d Grubbs outlier.

IDF levels ranged from 5.64 to 23.00% (glycerol IS; Table **2022.01A**) and 5.37 to 22.91% (DEG IS; Table **2022.01D**). SDF levels ranged from 7.41 to 33.25% (glycerol IS; Table **2022.01B**) and 7.64 to 34.94% (DEG IS; Table **2022.01E**). TDF levels ranged from 22.58 to 40.85% (glycerol IS; Table **2022.01C**) and 22.87 to 41.19% (DEG IS; Table **2022.01F**). Raw data for IDF, SDF, and TDF from the collaborative study are shown in Tables 3 and 4.

The S_r_, S_R_, RSD_r_, and RSD_R_ for IDF, SDF, and TDF with glycerol IS are shown in Tables 2022.01A–C and with DEG IS in Tables 2022.01D–F. For IDF, the values with the glycerol and DEG IS are very similar as would be expected. The small difference in values is due to the fact that not all laboratories included both IS in the samples being analyzed; Laboratory 2 did not include the glycerol IS and Laboratories 1 and 3 did not include the DEG IS. Laboratory 11 included the DEG IS for just half of the samples analyzed. Values for S_r_, S_R_, RSD_r_, and RSD_R_ for these fractions are in a similar range as those reported for AOAC Method **2011.25** [Reference (4), Table 1A]. With the glycerol IS, expanded measurement uncertainty (*MU*) in measurement of IDF ranged from 0.79 to 3.99%, whereas with the DEG IS expanded *MU* ranged from 0.70 to 3.86%. For SDF, there is a significant difference in SDFS values obtained with either the glycerol or the DEG IS. This difference is a result of the presence of glycerol in the sample, and the effect this has on the determined value of SDFS (Table 5). However, for this effect to be of any significance, the sample must have a glycerol content of >0.6 g/100 g. For example, with sample G/H with a glycerol content of 0.6 g/100 g, there is a moderate decrease in the determined SDFS value (approximately 0.6%) over the value obtained with the DEG IS. However, for sample K/N with a glycerol content of 2.23 g/100 g, a dramatic decrease in the measured SDFS content is observed, with a value of 23.44 g/100 g with the DEG IS and a value of 13.36 with the glycerol IS. Sample K/N is a high-fiber nutrition bar into which significant quantities of glycerol have been added, possibly as an anti-staling agent and to help maintain texture. The S_r_, S_R_, RSD_r_, and RSD_R_ for SDF with glycerol IS are shown in Table **2022.01B** and with DEG IS in Table **2022.01E**. With the DEG IS, s_r_ for SDF ranged from 0.41 to 0.84 g/100 g, and s_R_ ranged from 0.78 to 2.94 g/100 g. RSD_R_ ranged from 7.56 to 12.08%. Expanded *MU* ranged from 1.56 to 5.88%. Similar values were obtained with the glycerol IS. For TDF analysis with the DEG IS, s_r_ ranged from 0.59 to 1.35 g/100 g, and s_R_ ranged from 1.11 to 3.05 g/100 g. RSD_R_ ranged from 4.55 to 9.26%. Expanded *MU* ranged from 2.21 to 6.10%. Again, similar values for TDF were obtained with the glycerol IS.

**Table 5.** Comparison of the determined values of SDFS and TDF for samples using glycerol and DEG as the internal standard for HPLC

Sample^a^	Glycerol Internal Std		DEG Internal Std		Glycerol g/100 g
	SDFS g/100 g	TDF g/100 g	SDFS g/100 g	TDF g/100 g	
A & F	2.50	27.07	2.68	27.33	0.07
B & J	5.72	22.58	5.31	22.87	0.18
C & M	18.26	31.34	18.99	31.60	0.21
D & L	0.55	24.19	0.58	23.89	0.19
E & P	0.86	23.11	0.98	23.24	0.98
G & H	13.27	40.85	13.90	41.19	0.60
I & O	25.60	40.44	26.79	40.40	0.47
K & N	13.36	26.25	23.44	37.07	2.23

a A & F = Kidney beans; B & J = Rye crispbread; C & M = Chocolate; D & L = Carrots. E & P = Oat bran; G & H = Barley MAX flour; I & O = Miso soup powder; K & N = Nutrition bar (Fiber 1).

Repeatability, reproducibility, and expanded *MU* values were within the range of performance characteristics typically found for similar methods or similar analytical formats [Table 2 and Table **2011.25A–C** (5)].

DEG is a better IS than glycerol because on chromatography on TSK columns, there is a clear delineation of it from glycerol, oligosaccharides, and apparently all other components in the sample extract. Previously, glycerol had been chosen over DEG because of a perceived lesser stability of aqueous solutions of DEG over time. However, our recent studies have shown that aqueous solutions of DEG, in the presence or absence of glucose, are stable for at least 6 months at room temperature (in the dark), which is an acceptable period of stability to be of value in an analytical laboratory. Consequently, DEG is recommended as the preferred IS.

### Statistical Treatment

Collaborating laboratory data were evaluated statistically according to AOAC protocols using AOAC software. The raw data and statistically paired data from the blind duplicate results for IDF, SDF, and TDF with either the glycerol or DEG IS reported by the collaborating laboratories are shown in Tables 3a–c, 4a–c, **2022.01A–C** and **2022.01D–F**. Outliers and the reason for outlier removal are indicated and footnoted in Tables 3a–c, 4a–c.

Measurement uncertainty (14) of the insoluble, soluble and TDF procedure was assessed and calculated in accordance with the guidelines specified by Eurachem CITAC Guide CG4 (QUAM:2012.P1). The use of the s_R_ derived from a collaborative study as a measure of the combined standard uncertainty of the method is appropriate on the basis that: this is an empirical method whereby the measurement is dependent on the method used, there is no available certified reference material (CRM), and the method has been subject to a collaborative study whereby laboratory collaborators performed all stages of the method. Expanded *MU* is calculated using a coverage factor (*k*) of 2 on the basis that all mean results have a degrees of freedom greater than 25. The coverage factor (*k*) of 2 provides a level of confidence of approximately 95%.

The method was applied to collaborative study using eight sample matrixes (16 homogeneous test samples as 8 blind duplicates) analyzed by 17 collaborators.

The TDF value of a given sample is the sum of total of SDF (SDFS plus SDFP) and IDF. Raw data for IDF, SDF and TDF reported by the collaborating laboratories are shown in Tables 3a–c (glycerol IS ) and Tables 4a–c (DEG IS). Extremely high or low RSD_R_ values for IDF can be explained either by the fact that the measurement values were low (samples C/M and I/O), or by the fact that the sample contained carbohydrate material that was sparingly soluble in water (sample I/O) and values obtained were influenced by the severity of extraction (shaking/stirring). This is particularly evident with sample I/O (Miso soup) which contains seaweed polysaccharides.

## Discussion and Comments from Collaborators

In the procedure employed for the measurement of IDF, SDFS, SDFP, and TDF described in AOAC **2022.01**, there are several steps that must be performed as described to obtain correct values. In the incubation of PAA/AMG with samples, the enzyme employed, pH, temperature, and time of incubation are critical. However, there is some flexibility in the method concerning agitation during incubation with the proviso that the sample must be completely suspended during the full incubation period. The recommended procedure is to either use continual orbital shaking in a temperature-controlled bath or to continually stir the samples with an immersible magnetic stirrer. Laboratories 14 and 17 used linear shaking of incubation bottles attached at 45° angle to the direction of shaking while Laboratories 5, 6, 9, and 16 used ANKOM TDF equipment to achieve sample mixing. In most cases, insoluble fractions were recovered by filtration through Celite or using the ANKOM filtration system. One laboratory (6) used centrifugation to recover residues. In the analysis of the practice samples, glycerol was employed as the IS. However, because glycerol is found in various food samples at varying levels, several collaborating laboratories routinely employ DEG as the IS. The presence of significant levels of glycerol in samples leads to erroneously low values for the SDFS fraction. Consequently, in the analysis of the MLV samples, all collaborating laboratories, except Laboratories 1, 2, and 3 included both glycerol and DEG IS. Laboratories 1 and 3 used only glycerol and Laboratory 2 used only DEG. Because Laboratory 7 had initiated the analysis of the MLV samples before the decision was made to include the DEG as well as the glycerol IS, half of the results reported by them did not include DEG data. Two laboratories (6 and 11) analyzed residual protein using the Dumas method, and one of these labs (laboratory 11) analyzed a single sample and divided the residue for determination of both ash and protein. Laboratory 17 noted that in recovering the SDFP fraction from samples I and O, a sticky residue was present on the bottom of the bottles, and this was difficult to recover quantitatively. This sample (Miso soup powder) contains Wakame seaweed, which in turn contains carrageenan polysaccharides which are gelatinous and sparingly soluble in water. The nature of this polysaccharide most likely explains the very high RSD_r_ and RSD_R_ for the IDF fraction of this sample. One collaborator recommended that the washings in step **H(d)** be combined with the filtrate **H(c)** to ensure 100% recovery of the SDFS fraction. In the method described here, this is not important as the amount of SDFS is quantitated using the ratio of the areas of the SDFS fraction and DEG or glycerol IS. To ensure 100% recovery of the SDFS fraction the method was modified to include the washings **H(d)** with the SDFS fraction **H(c)**. Another collaborator highlighted the fact that several disaccharides and other components, like lactose, elute on TSK HPLC at various points between maltose and the SDFS fraction. These compounds include lactitol, maltitol, and isomalt (6-*O*-α-D-glucopyranosyl-D-glucitol mixed with 1-*O*-α-D-glucopyranosyl-D-mannitol). A method has been developed to remove lactose and isomaltose in the sample to allow clearer delineation between disaccharide and SDFS. Clearly, if these other compounds find significant use in food products at levels that might interfere with accurate measurement of SDFS, then procedures, possible enzymatic, could be developed to remove these compounds.

## Conclusions

AOAC **2009.01** is recognized by the Codex Alimentarius, the US Food and Drug Administration, and food authorities worldwide as the reference method for measuring TDF in foods and food ingredients. AOAC **2017.16** was developed and validated to address problems that were identified when applying AOAC **2009.01** to the measurement of fiber in specific fiber ingredients. Currently, AOAC 2017.16 is replacing AOAC 2009.01 as the Codex reference method for TDF. AOAC **2011.25** is a modification of AOAC **2009.01**, which allows for separate measurements of IDF and SDF (SDFP + SDFS). A similar modification has been made to AOAC **2017.16** to allow the separate measurement of IDF and SDF. This method, AOAC **2022.01** has been subjected to interlaboratory validation in the current study. Based on the results obtained from this study, as detailed herein, it is recommended that AOAC Method **2022.01** for the measurement of IDF, SDF, and TDF be adopted as a First Action Official Method.
